# Prethermalization for Deformed Wigner Matrices

**DOI:** 10.1007/s00023-024-01518-y

**Published:** 2024-12-17

**Authors:** László Erdős, Joscha Henheik, Jana Reker, Volodymyr Riabov

**Affiliations:** 1 Institute of Science and Technology Austria, Am Campus 1, 3400 Klosterneuburg, Austria; 2 ENS de Lyon UMPA, 46 Allée d’Italie, 69007 Lyon, France

**Keywords:** 60B20, 82C10

## Abstract

We prove that a class of weakly perturbed Hamiltonians of the form $H_\lambda = H_0 + \lambda W$, with *W* being a Wigner matrix, exhibits *prethermalization*. That is, the time evolution generated by $H_\lambda$ relaxes to its ultimate thermal state via an intermediate prethermal state with a lifetime of order $\lambda ^{-2}$. Moreover, we obtain a general relaxation formula, expressing the perturbed dynamics via the unperturbed dynamics and the ultimate thermal state. The proof relies on a two-resolvent law for the deformed Wigner matrix $H_\lambda$.

## Introduction

It is well-known (see, e.g., [24]) that certain macroscopic observables in an isolated quantum system with many interacting degrees of freedom tend to equilibrate, i.e., their expectation values become essentially constant at large times. However, if the system is coupled to the environment (reservoir), then the process of relaxation to equilibrium may take different forms depending on the properties of the initial system and the structure of the perturbation.

In this work, we consider a weakly coupled system of the form

1.1 $$\begin{aligned} H_\lambda := H_0 + \lambda W, \end{aligned}$$

where $H_0$ is a single-body or a many-body *Hamiltonian*, *W* is an energy preserving (Hermitian) *perturbation*, and $\lambda$ is a small coupling constant. For our phenomenological study, we consider a mean-field random perturbation that couples all modes. Following the extensive physics literature [15, 17–19, 30, 34], we choose the perturbation *W* to be a *Wigner random matrix*, i.e., a random matrix with centered, independent identically distributed (i.i.d.) entries (modulo the Hermitian symmetry).

The central object of our study is the perturbed time evolution of the quantum expectation value

1.2 $$\begin{aligned} \langle A \rangle _{P_\lambda (t)}:= \textrm{Tr}[P_\lambda (t) A] \end{aligned}$$

of an *observable* *A*, compared to the unperturbed evolution $\langle A \rangle _{P_0(t)} = \textrm{Tr}[P_0(t) A]$, which is considered known. Here

1.3 $$\begin{aligned} P_\lambda (t):=\textrm{e}^{-\textrm{i}t H_\lambda }P\textrm{e}^{\textrm{i}t H_\lambda } \qquad \text {resp.} \qquad P_0(t):=\textrm{e}^{-\textrm{i}t H_0}P\textrm{e}^{\textrm{i}t H_0} \end{aligned}$$

denote the Heisenberg time evolution of an initial *state* *P* governed by the (un)perturbed Hamiltonian. We point out that the *unperturbed* evolution strongly depends on all its constituents and hence, it might exhibit qualitatively different and generally complex behavior.

In contrast, the *perturbed* system relaxes to equilibrium via a robust mechanism, and it can be described by a fairly simple general *relaxation formula*

1.4 $$\begin{aligned} \langle A\rangle _{P_\lambda (t)}\approx \langle A\rangle _{\widetilde{P}_\lambda }+|g_\lambda (t)|^2 \big [\langle A\rangle _{P_0(t)}-\langle A\rangle _{\widetilde{P}_\lambda }\big ], \qquad g_\lambda (t): = \textrm{e}^{-\alpha \lambda ^2t}, \quad \alpha >0,\nonumber \\ \end{aligned}$$

where $\widetilde{P}_\lambda$ is the thermal state of the composite system (1.1). In this form, Eq. (1.4) is first mentioned in [30, Eq. (40)], where it describes the time dependence of the expectation of an observable in a nonintegrable system after perturbation by a random matrix.

The relaxation formula (1.4) shows convergence to the thermal state at an exponential rate on time scales of order $\lambda ^{-2}$ (in agreement with *Fermi’s golden rule*), but it also carries more refined information about the role of the unperturbed dynamics in the process. A particularly interesting case occurs if both the perturbed and unperturbed systems equilibrate but do not approach the same limiting value. This often happens if $H_0$ has an additional symmetry (conserved quantity) that is broken by the perturbation. If the time scale $\lambda ^{-2}$ of the perturbed equilibration is smaller than that of the unperturbed one, then the former robust process eclipses the latter. In particular, the precise form of $\langle A\rangle _{P_0(t)}$ in (1.4) is irrelevant whenever the prefactor $|g_\lambda (t)|^2$ is already exponentially small. In the opposite case, however, the equilibration of the perturbed dynamics happens in two stages. This phenomenon, known as *prethermalization* in the physics literature, was first described in a paper by Moeckel and Kehrein [28]. We remark, however, that this terminology was already used to describe a different phenomenon a few years earlier [6].

Nowadays, prethermalization has been extensively studied both experimentally (see, e.g., the review [26]) and theoretically (e.g., in [8, 20, 23, 25, 27, 31, 33], see also the review [29]). Reimann and Dabelow [15] studied the first relaxation stage of a prethermalization process, which is governed by $H_0$. More precisely, assuming that $P_0(t)$ relaxes to a non-thermal steady state, they find that the perturbed time evolution $\langle A\rangle _{P_\lambda (t)}$ with a sufficiently weak perturbation ($\lambda \ll 1$) closely follows the unperturbed time evolution $\langle A\rangle _{P_0(t)}$ for times $t \ll \lambda ^{-2}$. In particular, the perturbed time evolution $\langle A\rangle _{P_\lambda (t)}$ is close to the non-thermal steady state of $H_0$ for times $1 \ll t \ll \lambda ^{-2}$.^1^ The authors of [15] further extended their principal approach to a general study of relaxation theory for perturbed quantum dynamics in [17, 18]. These works now include all times and also the *strong* coupling regime (in case of banded matrices), which yields a characteristic power-law time decay (given more precisely by a Bessel function) instead of the exponential decay in (1.4). The theoretical model is then applied to several examples and compare the prediction to numerical and experimental works (see also Dabelow’s PhD thesis [14] for further details). Finally, we also mention that prethermalization in the form of the existence of an effectively conserved quantity for very long times has been rigorously established in [1] for periodically driven quantum systems if the frequency is large compared with the size of the driving potential.

In this paper, we approach prethermalization from the viewpoint of random matrix theory, interpreting the unperturbed Hamiltonian $H_0 \equiv H_0(N) \in \textbf{C}^{N \times N}$ as a fixed sequence of bounded self-adjoint deterministic matrices and the perturbation $W\equiv W(N)$ as an $N\times N$ Wigner random matrix. Our Hamiltonian $H_\lambda$ in the setting of (1.1) is also called *deformed Wigner matrix* in random matrix theory, or it can be viewed as a Wigner random matrix with nonzero expectation. Wigner matrices are encountered in many related physics models, e.g., the recent rigorous study of thermalization problems [11–13]. Here, the key technical result is a strong concentration property of the resolvent $G(z)= (H_\lambda -z)^{-1}$ or products of several resolvents around their deterministic approximation. Such results are commonly called *multi-resolvent global or local laws*, depending on the distance of the spectral parameter from the spectrum. For example, a typical *two-resolvent* law computes1.5to leading order in *N*, where  denotes the normalized trace, $z_1,z_2\in \textbf{C}\setminus \textbf{R}$, and $A_1,A_2\in \textbf{C}^{N\times N}$ are deterministic matrices. Using functional calculus, the resolvents can be replaced by more general and even *N*-dependent functions, thus linking (1.5) to the Heisenberg time evolution. Recent work [9] establishes a multi-resolvent local law for deformed Wigner matrices in the bulk regime of the spectrum, which motivated our study of perturbed quantum systems.

### Description of the Main Results

The principal goal of this work is a rigorous proof of the relaxation formula (Corollary 2.7) and prethermalization (Corollary 2.10) for perturbed quantum Hamiltonians of the form (1.1). We thereby assume that the unperturbed Hamiltonian $H_0$ has a (locally) regular limiting density of states $\rho _0$ around a reference energy $E_0$ and only energies in a microscopically large but macroscopically small interval $I_\Delta := [E_0 - \Delta , E_0+ \Delta ]$ are populated by the initial state *P* (similar assumptions are made in [15, 17, 18]). We then show the following corollaries of our main Theorem 2.4:

Cor. 2.7: The relaxation formula (1.4) holds generally for short and long kinetic times, i.e. $t \ll \lambda ^{-2}$ and $t \gg \lambda ^{-2}$, corresponding to $|g_\lambda (t)|^2 \approx 1$ and $|g_\lambda (t)|^2 \approx 0$, respectively. At intermediate times, $t \sim \lambda ^{-2}$ it is generally *not* valid, unless the quadratic forms $\langle \varvec{u}_j, A \varvec{u}_j \rangle$ of overlaps with the eigenvectors $\varvec{u}_j$ of $H_0$ behave regularly in *j* (cf. Definition 2.6). This happens, e.g., if $H_0$ satisfies the eigenstate thermalization hypothesis (ETH).

**Fig. 1:**
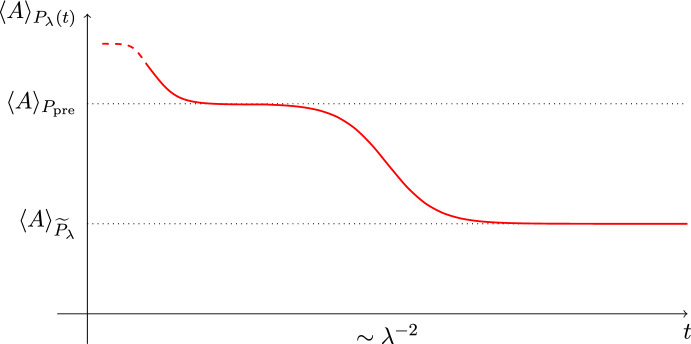
Depicted is a schematic graph of the prethermalization process: For times $1 \ll t\ll \lambda ^{-2}$, the perturbed time evolution of the quantum expectation $\langle A \rangle _{P_\lambda (t)}$ (see (1.2)) is close to the quantum expectation of *A* in the prethermal state $P_{\textrm{pre}}$, i.e. $\langle A \rangle _{P_\lambda (t)} \approx \langle A \rangle _{P_{\textrm{pre}}}$. For times $t \gg \lambda ^{-2}$, we have that $\langle A \rangle _{P_\lambda (t)}$ is close to the limiting thermal quantum expectation, i.e. $\langle A \rangle _{P_\lambda (t)} \approx \langle A \rangle _{\widetilde{P}_{\lambda }}$. This value is typically different from the prethermal quantum expectation $\langle A \rangle _{P_{\textrm{pre}}}$. This means, the ultimate relaxation of $\langle A \rangle _{P_\lambda (t)}$ toward $\langle A \rangle _{\widetilde{P}_{\lambda }}$ happens via an intermediate *prethermal* value $\langle A \rangle _{P_{\textrm{pre}}}$ in two steps, whose time scales are separated by $\lambda ^{-2}$

Cor. 2.10: Assuming that the unperturbed time evolution has a long time limit $\langle A \rangle _{P_0(t)} \overset{t \rightarrow \infty }{\longrightarrow }\ \langle A \rangle _{P_{\textrm{pre}}}$, such that the *prethermal state* $P_{\textrm{pre}}$ is distinguishable from the thermal state $\widetilde{P}_{\lambda }$ of the perturbed system, i.e. $\langle A \rangle _{P_{\textrm{pre}}} \ne \langle A \rangle _{\widetilde{P}_\lambda }$ (cf. Definition 2.9), we show the characteristic two-step relaxation of a prethermalization process; see Fig. 1.

### Outline of the Paper

**Fig. 2:**
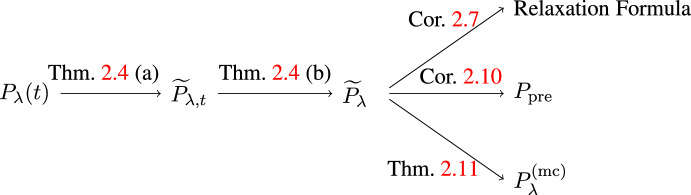
The structure of our main results

The general task in this paper is to approximately evaluate the random time evolution $\langle A \rangle _{P_\lambda (t)}$ from (1.2). This is carried out in several steps summarized schematically in Fig. 2. First, in Theorem 2.4 (a) in Sect. 2.2, the true time evolution $P_\lambda (t)$ is expressed as a linear combination of the unperturbed time evolution $P_0(t)$ and another deterministic time-dependent object $\widetilde{P}_{\lambda , t}$ that is conceptually simpler than $P_\lambda (t)$. Then, in Theorem 2.4 (b), we identify a time-independent state $\widetilde{P}_{\lambda }$ as the large time limit of $\widetilde{P}_{\lambda , t}$. Combining both parts of Theorem 2.4, we arrive at Corollary 2.7, which establishes the relaxation formula (1.4) at small and large kinetic times. As mentioned above, at intermediate times, it holds only for observables having the *local overlap regularity (LOR) property* (see Definition 2.6). In the subsequent Sect. 2.3, dropping the LOR property, but assuming additionally that the unperturbed Hamiltonian $H_0$ and the initial state *P* have the *prethermalization property* (see Definition 2.9), we obtain the characteristic two-scale relaxation of $P_\lambda (t)$ toward $\widetilde{P}_{\lambda }$ via an intermediate *prethermal state* $P_{\textrm{pre}}$ (see Corollary 2.10 and Fig. 1). As an additional result, in Theorem 2.11 in Sect. 2.4 we relate $\widetilde{P}_\lambda$ to the microcanonical ensemble of $H_\lambda$, called $P_\lambda ^\mathrm{(mc)}$, which is independent of the initial state *P*. Finally, our results are illustrated by two simple examples in Sect. 2.5.

While most proofs are given in Sect. 3, some auxiliary results and additional proofs are deferred to Appendix A.

### Notation

For positive quantities *f*, *g* we write $f\lesssim g$ (or $f=\mathcal {O}(g)$) and $f\sim g$ if $f \le C g$ or $c g\le f\le Cg$, respectively, for some constants $c,C>0$ which only depend on the constants appearing in the moment condition (see (2.1)) and the definition of the set of admissible energies (see (2.4)). In informal explanations, we frequently use the notation $f \ll g$, which indicates that *f* is “much smaller” than *g*. Moreover, we shall also write $w \approx z$ to indicate the closeness of $w, z \in \textbf{C}$ with a not precisely specified error.

For any natural number *n* we set $[n]: =\{ 1, 2,\ldots ,n\}$. Matrix entries are indexed by lowercase Roman letters *a*, *b*, *c*, ... and *i*, *j*, *k*, ... from the beginning or the middle of the alphabet and unrestricted sums over *a*, *b*, *c*, ... and *i*, *j*, *k*, ... are always understood to be over $[N] = \{1,...,N\}$.

We denote vectors by bold-faced lowercase Roman letters ${\varvec{x}}, {\varvec{y}}\in \textbf{C}^N$, for some $N\in \textbf{N}$. Vector and matrix norms, $\Vert {\varvec{x}}\Vert$ and $\Vert A\Vert$, indicate the usual Euclidean norm and the corresponding induced matrix norm. For any $N\times N$ matrices *A*, *B* we use the notations  to denote the normalized trace of *A* and $\langle A \rangle _B:= \textrm{Tr}[AB]$ is the trace of the product *AB*. We denote the spectrum of a matrix or operator *A* by $\sigma (A)$. Moreover, for vectors ${\varvec{x}}, {\varvec{y}}\in \textbf{C}^N$ and matrices $A\in \textbf{C}^{N\times N}$ we define

$$ \langle {\varvec{x}},{\varvec{y}}\rangle := \sum _{i} \overline{x}_i y_i\,, \qquad A_{\varvec{x} \varvec{y}}:= \langle \varvec{x}, A \varvec{y} \rangle \,. $$

For a unit vector $\varvec{v} \in \textbf{C}^{N}$ we shall also use the notation $\vert \varvec{v}\rangle \langle \varvec{v}\vert$ for the projection onto the one-dimensional subspace spanned by $\varvec{v}$.

Finally, we use the concept of “with very high probability” *(w.v.h.p.)* meaning that for any fixed $C>0$, the probability of an *N*-dependent event is bigger than $1-N^{-C}$ for $N\ge N_0(C)$. We introduce the notion of *stochastic domination* (see e.g. [21]): given two families of non-negative random variables

$$ X=\left( X^{(N)}(u): N\in \textbf{N}, u\in U^{(N)} \right) \quad \textrm{and}\quad Y=\left( Y^{(N)}(u): N\in \textbf{N}, u\in U^{(N)} \right) $$

indexed by *N* (and possibly some parameter *u* in some parameter space $U^{(N)}$), we say that *X* is stochastically dominated by *Y*, if for all $\xi , C>0$ we have

1.6 $$\begin{aligned} \sup _{u\in U^{(N)}} \textbf{P}\left[ X^{(N)}(u)>N^\xi Y^{(N)}(u)\right] \le N^{-C} \end{aligned}$$

for large enough $N\ge N_0(\xi ,C)$. In this case we use the notation $X\prec Y$ or $X= \mathcal {O}_\prec (Y)$.

## Main Results

In Sect. 2.1, we give the precise definition of Wigner random matrices and the assumptions on the Hamiltonian, observables and states under consideration in (1.1)–(1.3). Afterward, we formulate our main results in Sects. 2.2–2.4. Finally, in Sect. 2.5 we discuss our findings in the context of two simple examples.

### Assumptions

We begin with formulating the assumption on the Wigner matrix *W*.

#### Assumption 2.1

(Wigner matrix). Let $W \equiv W(N) = (w_{ij})_{i,j \in [N]}$ from (1.1) be a real symmetric or complex Hermitian random matrix $W = W^*$ with independent entries distributed according to the laws $w_{ij} {\mathop {=}\limits ^{\textrm{d}}} N^{-1/2}\chi _{\textrm{od}}$ for $i < j$ and $w_{jj} {\mathop {=}\limits ^{\textrm{d}}} N^{-1/2}\chi _{\textrm{d}}$. The random variables $\chi _{\textrm{od}}$ and $\chi _{\textrm{d}}$ satisfy the following assumptions^2^: We assume that $\chi _{\textrm{d}}$ is a centered real random variable, and $\chi _{\textrm{od}}$ is a real or complex random variable with $\textbf{E}\chi _{\textrm{od}} = 0$ and $\textbf{E}|\chi _{\textrm{od}}|^2 = 1$.

Furthermore, we assume the existence of higher moments, namely

2.1 $$\begin{aligned} \textbf{E}|\chi _{\textrm{d}}|^p + \textbf{E}|\chi _{\textrm{od}}|^p \le C_p, \end{aligned}$$

for all $p\in \textbf{N}$, where $C_p$ are positive constants.

For concreteness, we focus on the complex case with the additional assumptions $\textbf{E}\chi _{\textrm{od}}^2 = 0$ and $\textbf{E}|\chi _{\textrm{d}}|^2 = 1$; all other cases can also be handled as in [11]. The precise conditions on the Wigner matrix only play a role in the underlying *two-resolvent global law* (Proposition 3.1).

For the Hamiltonian $H_0 \equiv H_0(N)$ in (1.1) we assume the following.

#### Assumption 2.2

($H_0$ and its density of states). The Hamiltonian $H_0 \equiv H_0(N)$ is deterministic, self-adjoint $H_0 = H_0^*$, and uniformly bounded $\Vert H_0 \Vert \lesssim 1$. We denote the resolvent of $H_0$ at any spectral parameter $z \in \textbf{C}{\setminus } \textbf{R}$ by

$$\begin{aligned} M_0(z):= \frac{1}{H_0 -z}. \end{aligned}$$

Moreover, we assume the following:

(i) There exists a compactly supported measurable function $\rho _0: \textbf{R}\rightarrow [0,+\infty )$ with
  $$\int _\textbf{R}\rho _0(x) \textrm{d}x = 1$$
  and two positive sequences $\epsilon _0(N)$ and $\eta _0(N)$, both converging to zero as $N\rightarrow \infty$, such that, uniformly in $z \in \textbf{C}\backslash \textbf{R}$ with $\eta :=|\Im z| \ge \eta _0 \equiv \eta _0(N)$, we have 2.2 Here,
  2.3 $$\begin{aligned} m_0(z):= \int _\textbf{R}\frac{\rho _0(x)}{x-z}\textrm{d}x \end{aligned}$$
  is the Stieltjes transform of $\rho _0$. We refer to $\rho _0$ as the *limiting density of states*, and to $\textrm{supp}(\rho _0)$ as the *limiting spectrum* of $H_0$.
(ii) For small positive constants $\kappa ,c>0$, we define the set of *admissible energies* $\sigma _{\textrm{adm}}^{(\kappa ,c)}$ in the limiting spectrum of $H_0$ by^3^
  2.4 $$\begin{aligned} \sigma _{\textrm{adm}}^{(\kappa ,c)}:= \left\{ x \in \textrm{supp}(\rho _0): \inf _{|y-x|\le \kappa }\rho _0(y) > c,\, \Vert \rho _0\Vert _{C^{1,1}([x-\kappa , x+\kappa ])} \le 1/c\right\} . \nonumber \\ \end{aligned}$$
  We assume that for some positive $\kappa ,c> 0$, $\sigma _{\textrm{adm}}^{(\kappa ,c)}$ is not empty.

Assuming that the set of admissible energies in (2.4) is non-empty ensures that there is a part of the limiting spectrum $\textrm{supp}(\rho _0)$, where the limiting density of states $\rho _0$ behaves regularly, i.e. it is strictly positive and sufficiently smooth. Finally, we formulate our assumptions on the states considered in (1.2) and (1.3).

#### Assumption 2.3

(States). Given Assumption 2.2, we first pick a *reference energy*

2.5 $$\begin{aligned} E_0 \in \sigma _{\textrm{adm}}^{(\kappa _0,c_0)} \quad \text {for some} \quad \kappa _0, c_0>0, \end{aligned}$$

and further introduce $I_\delta :=[E_0-\delta ,E_0+\delta ]$ for any $0<\delta <\kappa _0$. Moreover, take an *energy width* $\Delta \in (0, \frac{1}{6}\kappa _0)$ and let $\Pi _\Delta := \mathbbm {1}_{I_\Delta }(H_0)$ be the spectral projection of $H_0$ onto the interval $I_\Delta$.

Then, we assume that the (deterministic) initial state $P \equiv P(N) \in \textbf{C}^{N \times N}$ in (1.3) is a state in the usual sense ($P = P^*$, $0 \le P \le 1$, and $\textrm{Tr}[P] = 1$), and is localized in $I_\Delta$, i.e.

2.6 $$\begin{aligned} P = \Pi _\Delta P \Pi _\Delta . \end{aligned}$$

Note that we assume only the state *P* to be localized in $I_\Delta$ and not the (deterministic) observable $A \equiv A(N) \in \textbf{C}^{N \times N}$. However, by inspecting the proof, we see that *A* and *P* play essentially symmetric roles, and thus, our results hold verbatim if we assume localization of *A* instead of *P*. Moreover, for ease of notation, we drop the *N*-dependence of all the involved matrices.

### Relaxation of Perturbed Quantum Dynamics

In this section, we present our main result on the time evolution of the random quantum expectation $\langle A \rangle _{P_\lambda (t)}$ from (1.2).

Its relaxation is described in two steps, hence Parts (a) and (b) in the following theorem. In the first step, we eliminate the randomness and identify the leading deterministic part of $\langle A \rangle _{P_\lambda (t)}$ in terms of $M_0$, the unperturbed resolvent. In the second step, we consider the short- and long-time limits of the leading term. Further explanatory comments come after the theorem and in Remark 2.5 below.

#### Theorem 2.4

(Relaxation of perturbed dynamics). Let $H_\lambda = H_0 + \lambda W$ be a perturbed Hamiltonian like in (1.1) with $\lambda > 0$, whose constituents satisfy Assumptions 2.1 and 2.2, respectively. Pick a reference energy $E_0$ like in (2.5). Let *P* be a state satisfying Assumption 2.3 for some energy width $\Delta > 0$ and *A* a bounded deterministic observable, $\Vert A \Vert \lesssim 1$.

Then, using the notations (1.2) and (1.3), we have the following two approximation statements:

- *[Relaxation in the kinetic limit]* The perturbed dynamics $\langle A \rangle _{P_\lambda (t)}$ satisfies
  2.7 $$\begin{aligned} \langle A \rangle _{P_\lambda (t)}=|g_\lambda (t)|^2 \langle A\rangle _{P_0(t)}+ \langle A \rangle _{\widetilde{P}_{\lambda ,t}}+ \mathcal {E}, \end{aligned}$$
  where we denoted
  2.8 $$\begin{aligned} g_\lambda (t):= \textrm{e}^{-\alpha \lambda ^2 t} \quad \text {with} \quad \alpha := \pi \rho _0(E_0) . \end{aligned}$$
  Moreover, we introduced^4^
  2.9 $$\begin{aligned} \widetilde{P}_{\lambda ,t}:= \frac{\int _\textbf{R}\int _\textbf{R}\Im M_0(x + \textrm{i}\alpha \lambda ^2) \, K_{\lambda , t}(x-y) \, \langle \Im M_0(y + \textrm{i}\alpha \lambda ^2) \rangle _P \, \textrm{d}x \textrm{d}y}{\int _\textbf{R}\textrm{Tr}[\Im M_0(x + \textrm{i}\alpha \lambda ^2)] \, \langle \Im M_0(x + \textrm{i}\alpha \lambda ^2) \rangle _P \, \textrm{d}x }, \end{aligned}$$
  with an explicit kernel given by
  2.10 $$\begin{aligned} K_{\lambda , t}(u):= \frac{1}{\pi }\frac{2 \alpha \lambda ^2 }{u^2 + (2 \alpha \lambda ^2)^2} \left( 2\alpha \lambda ^2 t \frac{\sin (tu)}{tu} - \cos (tu) + \textrm{e}^{-2\alpha \lambda ^2 t}\right) . \end{aligned}$$
  Finally, we have $\mathcal {E}= \mathcal {O}(\mathcal {E}_0) + \mathcal {O}_\prec (C(\lambda , t)/\sqrt{N})$ for some constant $C(\lambda , t) > 0$ and for every fixed $T \in (0, \infty )$, the deterministic error $\mathcal {E}_0=\mathcal {E}_0(\lambda , t, \Delta , N)$ satisfies
  2.11 $$\begin{aligned} \lim _{\Delta \rightarrow 0}\lim _{\begin{array}{c} t\rightarrow \infty \\ \lambda \rightarrow 0\\ \lambda ^2t=T \end{array}}\lim _{N\rightarrow \infty } \mathcal {E}_0=0. \end{aligned}$$
- *[Long and short kinetic time limit]* Defining
  2.12 $$\begin{aligned} \widetilde{P}_{\lambda }:= \frac{\int _\textbf{R}\Im M_0(x + \textrm{i}\alpha \lambda ^2) \, \langle \Im M_0(x + \textrm{i}\alpha \lambda ^2) \rangle _P \, \textrm{d}x }{\int _\textbf{R}\textrm{Tr}[\Im M_0(x + \textrm{i}\alpha \lambda ^2)] \, \langle \Im M_0(x + \textrm{i}\alpha \lambda ^2) \rangle _P \, \textrm{d}x }, \end{aligned}$$
  it holds that
  2.13 $$\begin{aligned} \langle A \rangle _{\widetilde{P}_{\lambda ,t}} = \big (1 - |g_\lambda (t)|^2\big ) \langle A \rangle _{\widetilde{P}_{\lambda }} + \mathcal {R}, \end{aligned}$$
  where, for every fixed $T \in (0,\infty )$, the error term $\mathcal {R} = \mathcal {R}(\lambda , t, \Delta , N)$ satisfies
  2.14 $$\begin{aligned} \begin{aligned} \limsup _{\Delta \rightarrow 0} \limsup _{\begin{array}{c} t\rightarrow \infty \\ \lambda \rightarrow 0\\ \lambda ^2 t=T \end{array}} \limsup _{N\rightarrow \infty } |\mathcal {R}| \lesssim T\textrm{e}^{-2\alpha T}. \end{aligned} \end{aligned}$$

We point out that the error $\mathcal {E}$ in (2.7) naturally consists of two parts, a deterministic and a stochastic one. The stochastic part of order $\mathcal {O}_\prec (C(\lambda , t)/\sqrt{N})$ is obtained from a *global law* for two resolvents of the random matrix $H_\lambda$ (see (3.6) below); the deterministic part $\mathcal {O}(\mathcal {E}_0)$ is obtained from estimating the deterministic leading term in (3.7).

Note that the error $\mathcal {R}$ is small compared to the first term in the rhs. of (2.13) only in the regime where *T* is large, in particular $\langle A \rangle _{\widetilde{P}_{\lambda ,t}}$ converges to $\langle A \rangle _{\widetilde{P}_{\lambda }}$ exponentially fast. In the small *T* regime, both terms on the right-hand side of (2.13) vanish linearly in *T*. We chose the above formulation (2.13) because, in this way, it relates directly to the relaxation formula (1.4) (see Corollary 2.7 below).

#### Remark 2.5

We have two further comments on Theorem 2.4.

(i) The triple limits in (2.11) and (2.14) consist of a thermodynamic limit ($N\rightarrow \infty$), a kinetic limit or *van Hove limit* ($t\rightarrow \infty$ and $\lambda \rightarrow 0$ while $\lambda ^2t$ is fixed), and an infinitesimal spectral localization ($\Delta \rightarrow 0$). Note that the *kinetic time parameter* $T=\lambda ^2t$ is natural, as the time scale prescribed for relaxation in the physics literature, e.g., by explicit analysis of the Pauli master equation in [29, Sect. 5.2.6], is $\mathcal {O}(\lambda ^{-2})$. The $\Delta \rightarrow 0$ limit is needed only to ensure that the mean level spacing is approximately constant near $E_0$ on the scale $\Delta$. If the density of states is flat, the $\Delta \rightarrow 0$ limit can be omitted. We emphasize that the error terms in Theorem 2.4 are explicit in the sense that their dependence on the scaling parameters *N*, *t*, $\lambda$, and $\Delta$ is tracked throughout the proof. The limit in (2.11) is then the natural order of limits in which these errors vanish. Finally, we remark that the explicitly tracked errors allow for certain combined limits, although for simplicity, we do not pursue these extensions.
(ii) The idea behind (2.12)–(2.14) is that the kernel (2.10) is an approximate delta function with $T=\lambda ^2t$-dependent magnitude. More precisely, its Fourier transform^5^
  $$\begin{aligned} \widehat{K}_{\lambda , t}(p) = \frac{1}{\sqrt{2 \pi }} \, \big (1 - \textrm{e}^{-2 \alpha \lambda ^2(t - |p|)}\big ) \, \mathbbm {1}(|p|\le t) \end{aligned}$$
  converges uniformly in compact intervals to a constant. That is, for every fixed $T \in (0,\infty )$ and for every compact set $\Omega \subset \textbf{R}$, we have
  $$\begin{aligned} \lim _{\begin{array}{c} t\rightarrow \infty \\ \lambda \rightarrow 0\\ \lambda ^2 t=T \end{array}} \sup _{p \in \Omega }\left| \widehat{K}_{\lambda , t}(p) - \frac{1}{\sqrt{2 \pi } } \big (1 - \textrm{e}^{-2\alpha T}\big )\right| = 0. \end{aligned}$$
  However, since $x \mapsto \Im M_0(x + \textrm{i}\alpha \lambda ^2)$ is only regular on scale $\lambda ^2$, the approximation
  $$K_{\lambda , t}(x-y) \approx \big (1 - \textrm{e}^{-2\alpha \lambda ^2 t}\big ) \delta (x-y),$$
  used in heuristically obtaining (2.13) from (2.9) and (2.12), is *not* generally valid unless *T* is very small or very large, as (2.14) indicates.

In the remaining part of the current Sect. 2.2, we connect Theorem 2.4 to the relaxation formula (1.4). As a preparation, we formulate the following *local overlap regularity property*, required in Corollary 2.7 (c) below. For this purpose, let $\mu _j$ and $\varvec{u}_j$ denote the eigenvalues and corresponding normalized eigenvectors of

2.15 $$\begin{aligned} H_0 = \sum _j \mu _j \vert \varvec{u}_j\rangle \langle \varvec{u}_j\vert . \end{aligned}$$

#### Definition 2.6

(Local overlap regularity (LOR)). Let the Hamiltonian $H_0$ be as in Assumption 2.2. We say that a bounded deterministic observable *A*, $\Vert A \Vert \lesssim 1$, has the *local overlap regularity (LOR) property* if and only if the eigenvector overlaps $\langle \varvec{u}_j, A \varvec{u}_j \rangle$ are approximately constant in the following sense: There exists a constant $\mathfrak {A} \in \textbf{R}$ such that^6^

2.16 $$\begin{aligned} \langle \varvec{u}_j, A \varvec{u}_j \rangle = \mathfrak {A} + \mathcal {O}\big (\mathcal {E}_{\textrm{LOR}}\big ) \quad \text {for all} \quad j \in \textbf{N}\quad \text {with} \quad \mu _j \in I_{2\Delta }, \end{aligned}$$

where the error $\mathcal {E}_{\textrm{LOR}} = \mathcal {E}_{\textrm{LOR}}(\Delta , N)$ satisfies

$$\begin{aligned} \lim _{\Delta \rightarrow 0}\lim _{N\rightarrow \infty } \mathcal {E}_{\textrm{LOR}} = 0. \end{aligned}$$

The LOR property (2.16) is satisfied, e.g., if $H_0$ satisfies the Eigenstate Thermalization Hypothesis [19, 32] (see also the discussion in [17]). For general systems, the ETH remains an unproven hypothesis. We remark, however, that it has been rigorously proven for a large class of mean-field random matrices $H_0$ (see [9, 11]), including Wigner matrices and their deformations.

The following corollary collects our rigorous results on the relaxation formula (1.4). The first two parts (items (a) and (b) below) immediately follow from Theorem 2.4 (a) and (b). The third part, item (c), involves the LOR property of the observable *A* and requires a separate argument, provided in Sect. 3.4.

#### Corollary 2.7

(Relaxation formula). Under the assumptions and using the notations of Theorem 2.4, it holds that

2.17 $$\begin{aligned} \langle A \rangle _{P_\lambda (t)} = \langle A \rangle _{\widetilde{P}_{\lambda }} + | g_\lambda (t)|^2 \left[ \langle A\rangle _{P_0(t)}- \langle A \rangle _{\widetilde{P}_{\lambda }}\right] + \mathcal {R} + \mathcal {E}. \end{aligned}$$

In particular, we have the following:

- *[Short kinetic time behavior]* Let $0<T\lesssim 1$. Then, it holds that
  2.18 $$\begin{aligned} \limsup _{\Delta \rightarrow 0} \limsup _{\begin{array}{c} t\rightarrow \infty \\ \lambda \rightarrow 0\\ \lambda ^2 t=T \end{array}} \limsup _{N\rightarrow \infty } \left| \langle A \rangle _{P_\lambda (t)} - \langle A\rangle _{P_0(t)}\right| \lesssim T \qquad \text {almost surely (a.s.) }\nonumber \\ \end{aligned}$$
- *[Long kinetic time behavior]* Let $T\gtrsim 1$. Then it holds that (recall $\alpha = \pi \rho _0(E_0)$)
  2.19 $$\begin{aligned} \limsup _{\Delta \rightarrow 0} \limsup _{\begin{array}{c} t\rightarrow \infty \\ \lambda \rightarrow 0\\ \lambda ^2 t=T \end{array}} \limsup _{N\rightarrow \infty } \left| \langle A \rangle _{P_\lambda (t)} - \langle A \rangle _{\widetilde{P}_{\lambda }}\right| \lesssim T \textrm{e}^{-2 \alpha T} \qquad \text {a.s. } \end{aligned}$$

Moreover, additionally assuming that *A* has the LOR property from Definition 2.6, we have:

(c) *[Intermediate kinetic times under LOR]* For every fixed $T \in (0,\infty )$ it holds that
  2.20 $$\begin{aligned} \lim _{\Delta \rightarrow 0}\lim _{\begin{array}{c} t\rightarrow \infty \\ \lambda \rightarrow 0\\ \lambda ^2t=T \end{array}}\lim _{N\rightarrow \infty }\big [ |\mathcal {R}| + |\mathcal {E}| \big ]= 0 \qquad \text {a.s. }, \end{aligned}$$
  i.e. the relaxation formula (1.4) is valid at *all* kinetic times $T \in (0,\infty )$.

Summarizing Corollary 2.7, we have that the relaxation formula (1.4) generally holds in the two limiting regimes (a) $|g_\lambda (t)|^2 \approx 0$ and (b) $|g_\lambda (t)|^2 \approx 1$, i.e. $T \ll 1$ or $T \gg 1$, respectively. In between, (1.4) is valid under the additional assumption that *A* has the LOR property from Definition 2.6, as this allows for the improved bound (2.20) on $\mathcal {R}$ compared to (2.14). However, *without* this regularity assumption, only the bound (2.14) (i.e. (2.18) and (2.19)) can hold, which indicates that the relaxation formula (1.4) is *not* generally valid for intermediate kinetic times $T \sim 1$. Indeed, it is easy to construct a counterexample. Finally, we remark that Corollary 2.7 (c) holds verbatim if the state *P* satisfies the LOR condition instead of the observable *A*. This simply follows by inspecting the proof in Sect. 3.4.

#### Remark 2.8

We have two further comments on Corollary 2.7.

(i) The relaxation formula (2.17) is in perfect agreement with the main result of Dabelow and Reimann, see [17, Eq. (16)]. In fact, the state $\widetilde{P}_\lambda$ defined in (2.12) agrees with $\widetilde{\rho }$ from [17, Eq. (16)], named the ” ’washed out’ descendant of the so-called diagonal ensemble” [17].
(ii) In fact, recalling (2.15), the proof of Theorem 2.4 (b) in Sect. 3.3 reveals that (see (3.71)) the error $\mathcal {R}$ in (2.13) and (2.17) is given by
  2.21 $$\begin{aligned} \mathcal {R} = \frac{1}{r} \sum _{j,k} \langle \varvec{u}_j, A \varvec{u}_j \rangle \langle \varvec{u}_k, P \varvec{u}_k \rangle \mathfrak {R}_{\lambda , t}(\mu _j - \mu _k), \end{aligned}$$
  where we denoted
  2.22 $$\begin{aligned} r:= \int _\textbf{R}\textrm{Tr}[\Im M_0(x + \textrm{i}\alpha \lambda ^2)] \, \langle \Im M_0(x + \textrm{i}\alpha \lambda ^2) \rangle _P \, \textrm{d}x \end{aligned}$$
  and
  2.23 $$\begin{aligned} \mathfrak {R}_{\lambda , t}(u):= \pi \textrm{e}^{-2\alpha \lambda ^2 t}\frac{2\alpha \lambda ^2}{u^2 + (2\alpha \lambda ^2)^2}\biggl (1-\cos (tu)-2\alpha \lambda ^2 t \frac{\sin (tu)}{tu}\biggr ). \end{aligned}$$
  The explicit error term (2.21)–(2.23) is in precise agreement with the error term in [17], see Eqs. (17) and (18). In particular, assuming the LOR property (2.16) for *A* (or *P*), the smallness of $\mathcal {R}$ in (2.20) for all kinetic times $T \in (0,\infty )$ is a consequence of the fact that $\int _\textbf{R}\mathfrak {R}_{\lambda , t}(u) \textrm{d}u = 0$.

### Prethermalization

In this section, we specialize the general relaxation theory of perturbed quantum dynamics from Theorem 2.4 to a class of unperturbed Hamiltonians $H_0$ and states *P* which *has the prethermalization property* in the following sense.

#### Definition 2.9

(Prethermalization property). Let the Hamiltonian $H_0$ and the state *P* be defined as in Assumptions 2.2 and 2.3. We say that $(H_0,P)$ has the *prethermalization property* if and only if there exists a state $P_{\textrm{pre}}$ (called the *prethermal state*) such that we have the following:

- The unperturbed time evolution $P_0(t)$ converges to $P_{\textrm{pre}}$, i.e., for all^7^ deterministic observables $A \in \textbf{C}^{N \times N}$ with $\Vert A \Vert \lesssim 1$, it holds that
  2.24a $$\begin{aligned} \lim _{t\rightarrow \infty } \lim \limits _{N \rightarrow \infty }\left[ \langle A\rangle _{P_0(t)}- \langle A \rangle _{P_{\textrm{pre}}}\right] = 0 . \end{aligned}$$
- There exists a bounded deterministic observable $A_0 \in \textbf{C}^{N \times N}$ which distinguishes $P_{\textrm{pre}}$ from $\widetilde{P}_\lambda$ (cf. (2.12)), i.e. there exists a constant $\mathfrak {c}_{\textrm{pre}}>0$ such that
  2.24b $$\begin{aligned} \liminf _{ \lambda \rightarrow 0} \liminf _{N \rightarrow \infty } \left| \langle A_0 \rangle _{P_{\textrm{pre}}} - \langle A_0 \rangle _{\widetilde{P}_\lambda } \right| \ge \mathfrak {c}_\textrm{pre}. \end{aligned}$$

We emphasize that $(H_0, P)$ having the *prethermalization property* is a purely deterministic condition, i.e., in particular, it does not depend on the Wigner matrix *W*. In the physics literature (see, e.g., [27] but also [5, 25, 26, 29]), the prethermalization property is generally expected to be satisfied if $H_0$ is an *integrable* Hamiltonian having at least one additional conserved quantity *Q* for which $[H_0, Q] = 0$.^8^ This symmetry is then broken by a generic perturbation *W*, i.e. $[W,Q] \ne 0$. In the presence of *M* conserved quantities $(Q_k)_{k=1}^M$, a good candidate for the prethermal state $P_{\textrm{pre}}$ is given by the so called *generalized Gibbs ensemble (GGE)*

$$ P_{\textrm{GGE}} = \frac{\textrm{e}^{- \sum _{k=1}^M \lambda _k Q_k}}{\textrm{Tr}\, \textrm{e}^{- \sum _{k=1}^M \lambda _k Q_k}}\,, $$

where the parameters $\lambda _k$ are chosen in such a way that $\textrm{Tr}Q_k P_{\textrm{GGE}} = \textrm{Tr}Q_k P$ for all $k\in [M]$ (see, e.g., [25] and [29, Section 5.1]). Exemplary pairs $(H_0, P)$ and observables $A_0$ satisfying the conditions in Definition 2.9 are given in Sect. 2.5.

Assuming that $(H_0, P)$ has the prethermalization property, Theorem 2.4 reads as follows.

#### Corollary 2.10

(Prethermalization). Under the assumptions of Theorem 2.4, let further $(H_0,P)$ have the prethermalization property from Definition 2.9. Then, recalling the notations from Theorem 2.4, it holds that

2.25 $$\begin{aligned} \langle A \rangle _{P_\lambda (t)} = \langle A \rangle _{\widetilde{P}_{\lambda }} + | g_\lambda (t)|^2 \left[ \langle A\rangle _{P_{\textrm{pre}}}- \langle A \rangle _{\widetilde{P}_{\lambda }}\right] + \mathcal {R} + \mathcal {E}'. \end{aligned}$$

We have $\mathcal {E}' = \mathcal {O}(\mathcal {E}_0') + \mathcal {O}_\prec (C(\lambda , t)/\sqrt{N})$ for some constant $C(\lambda , t) > 0$ and for every fixed $T \in (0, \infty )$, the deterministic errors $\mathcal {E}_0'=\mathcal {E}_0'(\lambda , t, \Delta , N)$ and $\mathcal {R} = \mathcal {R}(\lambda , t, \Delta , N)$ satisfy

$$\begin{aligned} \lim _{\Delta \rightarrow 0}\lim _{\begin{array}{c} t\rightarrow \infty \\ \lambda \rightarrow 0\\ \lambda ^2t=T \end{array}}\lim _{N\rightarrow \infty } \mathcal {E}_0'=0 \qquad \text {and} \qquad \limsup _{\Delta \rightarrow 0} \limsup _{\begin{array}{c} t\rightarrow \infty \\ \lambda \rightarrow 0\\ \lambda ^2 t=T \end{array}} \limsup _{N\rightarrow \infty } |\mathcal {R}| \lesssim T\textrm{e}^{-2\alpha T}. \end{aligned}$$

We remark that the error term $\mathcal {E}_0'$ contributing in (2.25) consists of two parts, $\mathcal {E}_0' = \mathcal {E}_0 + \mathcal {E}_{\textrm{pre}}$, with $\mathcal {E}_0 = \mathcal {E}_0(\lambda , t, \Delta , N)$ from Theorem 2.4 and $\mathcal {E}_{\textrm{pre}} = \mathcal {E}_{\textrm{pre}}(t,N)$ being the (absolute value of the) error in (2.24a). Note that (2.25) in particular implies the following small and large *T* behaviors:

2.26 $$\begin{aligned} \begin{aligned} \limsup _{\Delta \rightarrow 0} \limsup _{\begin{array}{c} t\rightarrow \infty \\ \lambda \rightarrow 0\\ \lambda ^2 t=T \end{array}} \limsup _{N\rightarrow \infty }\left| \langle A\rangle _{P_\lambda (t)} - \langle A\rangle _{P_{\textrm{pre}}}\right|&\lesssim T \quad \text {for} \quad T \lesssim 1 \quad \text {{a.s.}}, \\ \text {and} \quad \ \limsup _{\Delta \rightarrow 0} \limsup _{\begin{array}{c} t\rightarrow \infty \\ \lambda \rightarrow 0\\ \lambda ^2 t=T \end{array}} \limsup _{N\rightarrow \infty }\left| \langle A\rangle _{P_\lambda (t)}-\langle A\rangle _{\widetilde{P}_{\lambda }} \right|&\lesssim T\textrm{e}^{-2\alpha T} \quad \text {for} \quad T \gtrsim 1 \quad \text {{a.s.}} \end{aligned} \end{aligned}$$

Moreover, (2.24b) ensures that $\langle A \rangle _{P_{\textrm{pre}}} \ne \langle A \rangle _{\widetilde{P}_\lambda }$ for at least one observable $A= A_0$, which, together with (2.26) establishes Fig. 1 as a schematic graph of a prethermalization process.

### Connection to the Microcanonical Ensemble

Under an additional regularity assumption on  we can relate the state $\widetilde{P}_\lambda$ from (2.12) to the microcanonical ensemble.

#### Theorem 2.11

(Microcanonical average). Under the assumptions of Theorem  2.4, let us further assume thatis a Lipschitz continuous map on $I_\Delta$ with Lipschitz constant $\textrm{Lip}_{I_\Delta } (h)$ bounded in the sense that

2.27 $$\begin{aligned} \limsup _{\Delta \rightarrow 0} \, \limsup _{\lambda \rightarrow 0} \, \limsup _{N \rightarrow \infty }\textrm{Lip}_{I_\Delta } (h) \lesssim 1. \end{aligned}$$

Then

2.28 $$\begin{aligned} \langle A\rangle _{\widetilde{P}_\lambda }= \langle A \rangle _{P^\mathrm{(mc)}_{\lambda }}+\mathcal {E}_{\textrm{mc}} \qquad \text {with} \qquad P^\mathrm{(mc)}_\lambda := \frac{\Im M_0(E_0+ \textrm{i}\alpha \lambda ^2)}{\textrm{Tr}[\Im M_0(E_0 + \textrm{i}\alpha \lambda ^2)]} ,\nonumber \\ \end{aligned}$$

where the error $\mathcal {E}_{\textrm{mc}}=\mathcal {E}_{\textrm{mc}}(\lambda , \Delta , N)$ satisfies

$$\begin{aligned} \lim _{\Delta \rightarrow 0}\lim _{\begin{array}{c} \lambda \rightarrow 0 \end{array}}\lim _{N\rightarrow \infty }\mathcal {E}_\textrm{mc}=0. \end{aligned}$$

We emphasize that $P^\mathrm{(mc)}_\lambda$ is completely independent of the initial state *P*. Moreover, as mentioned above and already indicated by the notation, we can interpret $\langle A \rangle _{P_\lambda ^\mathrm{(mc)}}$ from (2.28) as the microcanonical average of $H_\lambda$ at energy $E_0$. The reason underlying this interpretation is that for any normalized eigenvector $\varvec{v}_{\lambda }$ of $H_\lambda$ with eigenvalue $E_\lambda$ very close to $E_0$, it holds that,^9^

$$\begin{aligned} \langle \varvec{v}_{\lambda }, A \varvec{v}_{\lambda } \rangle \approx \frac{\textrm{Tr}[\Im M_0(E_0+ \textrm{i}\alpha \lambda ^2)A ]}{\textrm{Tr}[\Im M_0(E_0 + \textrm{i}\alpha \lambda ^2)]} =\langle A \rangle _{P^\mathrm{(mc)}_{\lambda }} . \end{aligned}$$

This means, $P^\mathrm{(mc)}_\lambda$ is a close effective approximation to the actual projection $\vert \varvec{v}_{\lambda }\rangle \langle \varvec{v}_{\lambda }\vert$ onto the eigenspace spanned by $\varvec{v}_{\lambda }$.

### Examples

In this section, we give two examples of physical settings where prethermalization occurs and connect them to our assumptions. Note that both examples are one-dimensional. However, the extension to higher dimensions is straightforward. Moreover, although we do not express the Hamiltonians below as matrices, both act on finite-dimensional Hilbert spaces and can hence naturally be represented as such.

#### Next-Nearest Neighbor Hopping

For $N \in \textbf{N}$ even, we consider the Laplacian-like Hamiltonian $H_0$ acting on functions $\psi \in \ell ^2(\textbf{Z}/(N\textbf{Z}))$ as

2.29 $$\begin{aligned} (H_0\psi )(x):=2\psi (x)-\psi (x-2)-\psi (x+2) \end{aligned}$$

where $x-2$ and $x+2$ are interpreted mod *N*. Note that $H_0$ is similar to the discrete Laplacian with periodic boundary condition but induces next-nearest neighbor hopping instead of nearest neighbor hopping. In particular, $H_0$ conserves parity in the sense that functions that are only supported on the even or odd points of $\textbf{Z}/(N\textbf{Z})$, respectively, remain invariant, and thus its spectrum has an additional twofold degeneracy. This corresponds to the conserved quantity *Q* being the projection onto the even sites; clearly $[H_0, Q]=0$. Similar to the routine computations done for the discrete Laplacian, one can readily check the following:

- The Hamiltonian $H_0$ is bounded, $\Vert H_0\Vert \lesssim 1$.
- Its spectrum is given by $\sigma (H_0) = \left\{ 2 (1 - \cos (2 p_j)): p_j = 2 \pi j /N \right\} _{j \in [N]} \subset [0,4]$.
- The limiting density of states as $N\rightarrow \infty$ evaluates to
  2.30 $$\begin{aligned} \rho _0(x)=\frac{1}{\pi \sqrt{x(4-x)}}\mathbbm {1}_{[0,4]}(x) \end{aligned}$$
  which is compactly supported and satisfies the regularity assumptions in Assumption 2.2 for *x* bounded away from 0 and 4.

In this setting, we fix *k* such that the eigenvalue $2(1 - \cos (2 p_k))$ satisfies $p_k\in (0,\pi /2)$. Now take $P:= \vert \varvec{u}_k\rangle \langle \varvec{u}_k\vert$ with $\varvec{u}_k$ being the normalized eigenvector of $H_0$ supported on the *even* sub-lattice corresponding to the eigenvalue $2(1 - \cos (2 p_k))$. By construction, for every bounded observable *A* we have

$$\begin{aligned} \langle A\rangle _{P_0(t)}= \langle A \rangle _{P} = \langle A \rangle _{P_{\textrm{pre}}}, \quad \text {for all} \quad t\ge 0, \end{aligned}$$

since $[P,H_0]=0$. Hence, the symmetry implies that $P_{\textrm{pre}} = P$. In particular, for $A:=\textbf{1}_{\textrm{odd}}$ being the identity operator on the *odd* sub-lattice, its prethermal value is given by $\langle A\rangle _{P_{\textrm{pre}}}=0$. Moreover, by spectral decomposition of $H_0 = \sum _j \mu _j \vert \varvec{u}_j\rangle \langle \varvec{u}_j\vert$, we obtain2.31which implies that $\langle A\rangle _{\widetilde{P}_\lambda }\ne \langle A\rangle _{P_{\textrm{pre}}}$ (recall the definition of $\widetilde{P}_\lambda$ in (2.12)) for $A = \textbf{1}_{\textrm{odd}}$. Hence, we deduce that $(H_0, P)$ has the prethermalization property from Definition 2.9.

#### Free Spinless Fermions on a Lattice

As our second example, we consider a model of spinless fermions in a periodic one-dimensional lattice of even length *N* (cf. [17, App. B]), which can be seen as a many-body analog of the first example (although with nearest neighbor hopping instead of next-nearest neighbor hopping). Let

2.32 $$\begin{aligned} H_0=\frac{1}{\sqrt{N}}\sum _j c_j^{\dagger }c_{j+1}+c_{j+1}^\dagger c_j, \end{aligned}$$

where $c_j^\dagger$ and $c_j$ denote the fermionic creation and annihilation operators at site *j*, and the summation indices are considered modulo *N*. Note that the Hamiltonian in (2.32) conserves the particle number. It is readily checked that $H_0$ admits a limiting density of states which is not compactly supported but has fast decaying (Gaussian) tails. As the regularity assumptions in Assumption 2.2 (ii) are satisfied, this example is still sufficiently close to our theory to be described by it reasonably well.

In this setting, pick $\psi _j$ as the orthonormal eigenfunctions of the discrete Laplacian describing nearest neighbor hopping with periodic boundary conditions (i.e. the analog of (2.29) with $\pm 1$ instead of $\pm 2$) corresponding to the eigenvalues $2(1 - \cos (p_j))$ with

$$\begin{aligned} p_j:=\frac{2 \pi j}{N},\quad \frac{j}{N} \in \Big [\frac{1}{8}, \frac{3}{8}\Big ] \cup \Big [\frac{5}{8}, \frac{7}{8}\Big ]. \end{aligned}$$

We then construct $P:=\vert \psi \rangle \langle \psi \vert$ as a rank$-1$ projection onto an eigenstate of $H_0$ by taking

$$\begin{aligned} \psi :=\bigwedge _{j}\psi _j \end{aligned}$$

as a Slater determinant of the *N*/2 one-particle wave functions $\psi _j$. This ensures that *P* satisfies Assumption 2.3, as the density of states (which is the same as (2.30)) is regular in such intervals. Noting that $[P,H_0]=0$, we obtain

$$\begin{aligned} \langle A\rangle _{P_0(t)}= \langle A \rangle _{P} = \langle A \rangle _{P_{\textrm{pre}}}, \quad \text {for all} \quad t\ge 0, \end{aligned}$$

for every bounded observable *A*. Hence, $P_{\textrm{pre}} = P$, similar to the first example. In particular, for ${A=\textbf{1}_{\mathcal {H}_{N/2}^{\perp }}}$ being the identity on the orthogonal complement of the *N*/2-particle sector of the Fock space, the prethermal value is given by $\langle A\rangle _{P_\textrm{pre}} = 0$. Moreover, by spectral decomposition of $H_0$, similarly to (2.31), we find that $\langle A\rangle _{\widetilde{P}_\lambda } \ne \langle A\rangle _{P_\textrm{pre}}$. Hence, we deduce that $(H_0, P)$ has the prethermalization property from Definition 2.9.

## Proofs

In this section, we provide the proofs of our main results formulated in Sect. 2. We begin by giving the proof of Theorem 2.4, which we organize in three steps:

(i) In Sect. 3.1, as the first step, we approximate the random time evolution $\langle A \rangle _{P_\lambda (t)}$ by a deterministic object, up to an error vanishing as $N \rightarrow \infty$ with very high probability. This is done using a suitable *global law* for two resolvents of the random matrix $H_\lambda$ (see Proposition 3.1 below).
(ii) The deterministic object resulting from Step (i) consists of two terms, a regular and a singular one. In Sect. 3.2, we evaluate these terms up to errors captured by $\mathcal {E}$ (see Proposition 3.2). This proves Theorem 2.4 (a).
(iii) As the third and final step in Sect. 3.3, we examine the behavior of the singular term in the limits $T \rightarrow 0$ and $T \rightarrow \infty$ for $T:= \lambda ^2 t$ (see Proposition 3.6). This proves Theorem 2.4 (b).

Afterward, we give the proofs of Corollary 2.7 and Theorem 2.11 in Sects. 3.4 and 3.5, respectively. The proof of Corollary 2.10 is immediate from Definition 2.9 and Theorem 2.4 and hence omitted.

### Step (i): Global Law

Let $\lambda >0$ and let $H_\lambda := D + \lambda W$ such that $D\in \textbf{C}^{N\times N}$ is a self-adjoint deterministic matrix with $\Vert D\Vert \lesssim 1$ and *W* is a Wigner matrix satisfying Assumption 2.1. We refer to $H_\lambda$ as a *deformed Wigner matrix*. It is well known [2–4, 22], that the random resolvent^10^$G_\lambda (z):= (H_\lambda -z)^{-1}$ of $H_\lambda$ at spectral parameter $z \in \textbf{C}\setminus \textbf{R}$ is very well approximated by a deterministic matrix $M_\lambda$, which is the unique solution to the *Matrix Dyson Equation (MDE)*^11^3.1In particular (see [22, Theorem 2.1]), for $\textrm{dist}(z, \sigma (D) + [-2\lambda , 2 \lambda ]) \ge c$ for some *N*-independent $c > 0$ and $\sigma (D) \subset \textbf{R}$ denoting the spectrum of *D*, and arbitrary deterministic matrix $B \in \textbf{C}^{N \times N}$ with $\Vert B\Vert \lesssim 1$, it holds that3.2For our purposes, it is not sufficient to approximate only a single resolvent in the sense of (3.2). Instead, we need to establish the deterministic approximation to $\langle \varvec{x}, G_\lambda (z_1) B G_\lambda (z_2) \varvec{y} \rangle$ with two deterministic vectors $\varvec{x}, \varvec{y}$. This is the content of the following proposition, the proof of which is given in Appendix 3.5.

#### Proposition 3.1

(Isotropic two-resolvent global law for deformed Wigner matrices). Let $\lambda > 0$ and let $H_\lambda := D + \lambda W$ be an $N\times N$ deformed Wigner matrix (as in Assumption 2.1) with a bounded self-adjoint deformation $D\in \textbf{C}^{N\times N}$. Pick $B\in \textbf{C}^{N\times N}$, a deterministic matrix with $\Vert B\Vert \lesssim 1$, deterministic vectors $\varvec{x},\varvec{y}\in \textbf{C}^N$ with $\Vert \varvec{x} \Vert =\Vert \varvec{y}\Vert =1$, and two spectral parameters $z_1,z_2\in \textbf{C}$ satisfying $\min _{i \in [2]} \textrm{dist}(z_i, \sigma (D) + [-2\lambda , 2 \lambda ])\ge c$ for some *N*-independent parameter $c > 0$. Denote further $G_{\lambda , i}:=G_\lambda (z_i)=(H_\lambda -z_i)^{-1}$. Then,3.3where we denoted $M_{\lambda , i}:= M_\lambda (z_i)$ with $M_\lambda (z) \in \textbf{C}^{N \times N}$ being the solution of (3.1). The positive constant $C(\lambda ,c)$ in (3.3) depends^12^ only on its arguments $\lambda$ and *c*.

We now apply Proposition 3.1 to resolvents $G_\lambda (z):= (H_\lambda -z)^{-1}$ of our concrete deformed Wigner random matrix $H_\lambda = H_0 + \lambda W$. For the proof of Theorem 2.4, we use (3.3) as follows: Applying residue calculus allows to rewrite $\langle A\rangle _{P_\lambda (t)}$ as the contour integral

**Fig. 3:**
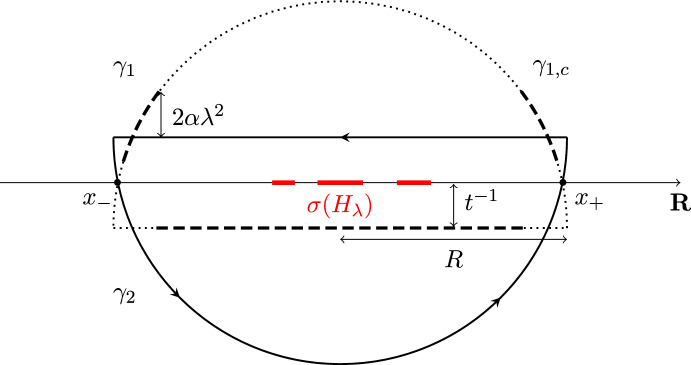
Sketch of the contours $\gamma _1$ and $\gamma _2$ from (3.4). The contour $\gamma _2$ is depicted as a solid black curve, while the contour $\gamma _1$ is indicated with dashed and dotted lines. The dotted parts of $\gamma _1$ constitute the set $\gamma _{1,c}$, defined in (3.32). The intersections of $\gamma _1$ (and $\gamma _2$) with the real line are denoted by $x_\pm$

3.4 $$\begin{aligned} \langle A\rangle _{P_\lambda (t)}  &   =\langle \textrm{e}^{-\textrm{i}t H_\lambda }P\textrm{e}^{\textrm{i}t H_\lambda }A\rangle \nonumber \\    &   = \frac{1}{(2 \pi \textrm{i})^2 }\oint _{\gamma _1}\oint _{\gamma _2}\textrm{e}^{\textrm{i}t(z_1-z_2)}\textrm{Tr}\big [ G_{\lambda }(z_1)AG_{\lambda }(z_2)P\big ]\textrm{d}z_1\textrm{d}z_2 \end{aligned}$$

where $\gamma _1$ and $\gamma _2$ are two semicircles (each being the complex conjugate of the other) with some large radius $R \gtrsim 1$ (see Fig. 3 below). We further define the contours such that the distance between the flat part of the semicircles and the real line is $t^{-1}$. Note that we have

3.5 $$\begin{aligned} \sigma (H_\lambda )\subseteq \sigma (H_0) + [-(2+\epsilon )\lambda , (2+\epsilon ) \lambda ]\quad \text {w.v.h.p.} \end{aligned}$$

for any fixed $\epsilon >0$ by standard perturbation theory, using that $\Vert W \Vert \le 2 + \epsilon$ with very high probability (see, e.g., [21, Theorem 7.6]). In particular, the contours encircle the spectrum of $H_\lambda$ completely if *R* is chosen large enough.

Writing $P=\sum _jp_j \vert \varvec{p}_j\rangle \langle \varvec{p}_j\vert$ in spectral decomposition and using that the $p_j \in [0,1]$ sum to one by Assumption 2.3, the global law (3.3) applied to ${\varvec{x}}={\varvec{y}}={\varvec{p}}_j$ implies^13^3.6uniformly for $z_1, z_2$ along the contours $\gamma _1, \gamma _2$ for any fixed $\lambda >0$. Just as in (3.3), $C(\lambda , t)$ denotes a positive constant depending only on $\lambda$ and *t*. Therefore, combining (3.4) with (3.6), we find that3.7To establish (2.7), our main task thus lies in evaluating the right-hand side of (3.7). For simplicity, we refer to the integrals in the first and second line of (3.7) as the *regular* and the *singular* term, respectively.

### Step (ii): Evaluation of the Regular and Singular Term and Proof of Theorem 2.4 (a)

We organize the result of our computation of (3.7) in the following proposition.

#### Proposition 3.2

(Evaluation of the regular and singular term). Under the assumptions of Theorem 2.4 and letting $\gamma _1$, $\gamma _2$ be the contours in Fig. 3, we have (recalling $\alpha = \pi \rho _0(E_0)$)

3.8a $$\begin{aligned} \begin{aligned}&\frac{1}{(2 \pi \textrm{i})^2 }\oint _{\gamma _1}\oint _{\gamma _2} \textrm{e}^{\textrm{i}t (z_1 - z_2)} \textrm{Tr}\big [M_{\lambda }(z_1)AM_{\lambda }(z_2)P \big ] \textrm{d}z_1\textrm{d}z_2 \\&=\textrm{e}^{-2 \alpha \lambda ^2t}\langle A\rangle _{P_0(t)}+\mathcal {O}(\mathcal {E}_\textrm{reg}), \end{aligned} \end{aligned}$$

for the regular term and3.8b for the singular term, with some error terms $\mathcal {E}_{\mathrm{reg/sing}} = \mathcal {E}_{\mathrm{reg/sing}}(\lambda , t, \Delta , N)$ in (3.8a) and (3.8b) satisfying (2.11). The explicit form of $\mathcal {E}_{\textrm{reg}}$ is given in (3.16) and (3.18), while the explicit form of $\mathcal {E}_{\textrm{sing}}$ is given in (3.26).

Plugging (3.8a) and (3.8b) into (3.7), we immediately conclude Theorem 2.4 (a) after setting $\mathcal {E}_0:= \mathcal {E}_{\textrm{reg}} + \mathcal {E}_{\textrm{sing}}$ and including the error term from (3.7) into $\mathcal {E}$. $\square$

It thus remains to give the proof of Proposition 3.2, i.e. its two parts (3.8a) and (3.8b). This is done in Sects. 3.2.1 and 3.2.2, respectively.

#### Proof of (3.8a)

The main contribution to the integral in (3.8a) comes from the regime^14^ where $z_1$ and $z_2$ are close to $E_0$. Hence, as a first approximation we use the replacements  and  in (3.1), which leads to

3.9 $$\begin{aligned} M_\lambda (z_1) \approx \frac{1}{H_0 -z_1 - \lambda ^2 \overline{m_0(E_0)}} \qquad \text {and} \qquad M_\lambda (z_2) \approx \frac{1}{H_0 -z_2 - \lambda ^2 {m_0(E_0)}}.\nonumber \\ \end{aligned}$$

Applying the replacements in (3.9) for the term in (3.8a) yields

3.10 $$\begin{aligned}  &   \frac{1}{(2 \pi \textrm{i})^2 }\oint _{\gamma _1}\oint _{\gamma _2} \textrm{e}^{\textrm{i}t (z_1 - z_2)}\textrm{Tr}\left[ \frac{1}{H_0 -z_1 - \lambda ^2 \overline{m_0(E_0)}}A\frac{1}{H_0 -z_2 - \lambda ^2 {m_0(E_0)}}P \right] \textrm{d}z_1\textrm{d}z_2 \nonumber \\  &   \quad = \textrm{Tr}\left[ \textrm{e}^{\textrm{i}t(H_0 - \lambda ^2 \overline{m_0(E_0)})}A \textrm{e}^{- \textrm{i}t(H_0 - \lambda ^2 {m_0(E_0)})} P \right] \nonumber \\  &   \quad = \textrm{e}^{-2 \Im m_0(E_0)\lambda ^2 t} \textrm{Tr}\left[ \textrm{e}^{\textrm{i}tH_0 }A \textrm{e}^{- \textrm{i}tH_0 } P \right] = \textrm{e}^{-2 \alpha \lambda ^2 t} \, \langle A \rangle _{P_0(t)}, \end{aligned}$$

since $\Im m_0(E_0) = \pi \rho _0(E_0)$, from simple residue calculus for $\lambda >0$ small enough, using that $|m_0(E_0)| \lesssim 1$ and $\gamma _1, \gamma _2$ encircle the spectrum of $H_0$. We have thus extracted the main term in (3.8a), and it remains to estimate the errors resulting from the replacements in (3.9).

Recall (see (2.15)) that $\mu _j$ and $\varvec{u}_j$ are the eigenvalues and the respective orthonormalized eigenvectors of $H_0$, i.e.

3.11 $$\begin{aligned} H_0 = \sum _j \mu _j \vert \varvec{u}_j\rangle \langle \varvec{u}_j\vert . \end{aligned}$$

Then, by means of  (3.1), spectral decomposition (3.11) of $H_0$ and using Assumption 2.3 together with $[H_0, \Pi _\Delta ] = 0$ and $\Pi _\Delta ^2 = \Pi _\Delta$, we have that

3.12 $$\begin{aligned} \text {lhs. of}\, 3.8a = \textrm{Tr}\big [\widetilde{\Theta }_1 A \widetilde{\Theta }_2 P\big ] = \sum _{\mu _i,\mu _j \in I_\Delta } \langle \varvec{u}_i, A \varvec{u}_j \rangle \langle \varvec{u}_j, P \varvec{u}_i \rangle \widetilde{\vartheta }_{1}(i) \widetilde{\vartheta }_{2}(j) , \nonumber \\ \end{aligned}$$

where we denoted

3.13 $$\begin{aligned} \widetilde{\Theta }_1:= \sum _{\mu _j\in I_\Delta } \vert \varvec{u}_j\rangle \langle \varvec{u}_j\vert \widetilde{\vartheta }_1(j) \quad \text {and} \quad \widetilde{\Theta }_2:= \sum _{\mu _j\in I_\Delta } \vert \varvec{u}_j\rangle \langle \varvec{u}_j\vert \widetilde{\vartheta }_2(j) \end{aligned}$$

with3.14Note that, by symmetry of the contours $\gamma _1$ and $\gamma _2$, we have that $\overline{\widetilde{\vartheta }_{1}(j)} = \widetilde{\vartheta }_{2}(j)$ and $\widetilde{\Theta }_1^* = \widetilde{\Theta }_2$.

The key to approximating (3.12) is the following lemma, whose proof is given at the end of the current Sect. 3.2.1.

##### Lemma 3.3

(First replacement lemma) Using the above notations and assumption, denote

$$\begin{aligned} \Theta _1:= \sum _{\mu _j \in I_\Delta } \vert \varvec{u}_j\rangle \langle \varvec{u}_j\vert \vartheta _1(j) \quad \text {with} \quad \vartheta _{1}(j):= \frac{1}{2 \pi \textrm{i}} \oint _{\gamma _1} \frac{\textrm{e}^{\textrm{i}t z_1}}{\mu _j - z_1 - \lambda ^2 \overline{m_0(E_0)}} \textrm{d}z_1 \end{aligned}$$

and $\Theta _2:= \Theta _1^*$ via $\vartheta _{2}(j):= \overline{\vartheta _{1}(j)}$, analogously to (3.13) and (3.14). Then it holds that

3.15 $$\begin{aligned} \sup _{\mu _i \in I_\Delta } \left| \widetilde{\vartheta }_{1}(i) - \vartheta _1(i) \right| + \sup _{\mu _j \in I_\Delta } \left| \widetilde{\vartheta }_{2}(j) - \vartheta _2(j) \right| \lesssim \widetilde{\mathcal {E}}_{\textrm{reg}} \end{aligned}$$

for sufficiently small $\lambda > 0$ and *N* large enough (dependent on $\lambda$, cf. Lemma A.1). Here, recalling (2.2) for the definition of $\epsilon _0$, we denoted

3.16 $$\begin{aligned} \widetilde{\mathcal {E}}_{\textrm{reg}}:= \lambda ^2 t \, \Delta + \lambda \, (1 + \lambda ^2 t)+ \frac{\lambda }{\Delta }\left( 1 + \frac{\lambda }{\Delta }\right) + \lambda ^2 t \, \epsilon _0. \end{aligned}$$

Therefore, by writing $\widetilde{\Theta } = \Theta + (\widetilde{\Theta } - \Theta )$ in (3.12), we find the lhs. of (3.8a) to be given by

3.17 $$\begin{aligned}  &   \textrm{Tr}\big [\Theta _1 A {\Theta }_2 P\big ] + \textrm{Tr}\big [(\widetilde{\Theta }_1- \Theta _1) A \Theta _2 P\big ] + \textrm{Tr}\big [\Theta _1 A (\widetilde{\Theta }_2-\Theta _2) P\big ]\nonumber \\    &   \quad + \textrm{Tr}\big [(\widetilde{\Theta }_1 - {\Theta }_1) A (\widetilde{\Theta }_2-{\Theta }_2) P\big ]. \end{aligned}$$

The first term in (3.17) precisely yields the result of (3.10) using Assumption 2.3. Using $\Vert A \Vert \lesssim 1$ and $\textrm{Tr}[P] = 1$, the second and third term in (3.17) can be estimated by (a constant times)

$$\begin{aligned} \Vert \Theta _1 \Vert \, \Vert \widetilde{\Theta }_2 - \Theta _2 \Vert + \Vert \widetilde{\Theta }_1 - \Theta _1 \Vert \, \Vert \Theta _2 \Vert \lesssim \widetilde{\mathcal {E}}_{\textrm{reg}}. \end{aligned}$$

Here we used (3.15) and that $\Vert \Theta _1 \Vert \le 1$ and $\Vert \Theta _2 \Vert \le 1$ as follows by the explicit expressions

$$\begin{aligned} \Theta _1 = \textrm{e}^{\textrm{i}t(\Pi _\Delta H_0 \Pi _\Delta - \lambda ^2 \overline{m_0(E_0)})} \quad \text {and} \quad \Theta _2 = \textrm{e}^{-\textrm{i}t(\Pi _\Delta H_0 \Pi _\Delta - \lambda ^2 {m_0(E_0)})}\, \end{aligned}$$

and $\Im m_0(E_0)\ge 0$. Similarly, applying (3.15) again, the fourth term in (3.17) is bounded by $\mathcal {O}(\widetilde{\mathcal {E}}_{\textrm{reg}}^2)$. Collecting all four terms of (3.16), this concludes the proof of (3.8a) with

3.18 $$\begin{aligned} \mathcal {E}_{\textrm{reg}}:= \widetilde{\mathcal {E}}_{\textrm{reg}} + \widetilde{\mathcal {E}}_\textrm{reg}^2. \end{aligned}$$

It remains to give the proof of Lemma 3.3.

##### Proof of Lemma 3.3

Since $\overline{\widetilde{\vartheta }_{1}(j)} = \widetilde{\vartheta }_{2}(j)$ and $\vartheta _{2}(j):= \overline{\vartheta _{1}(j)}$, we only estimate $\widetilde{\vartheta }_2(j)- \vartheta _2(j)$ for arbitrary but fixed index *j* such that $\mu _j \in I_\Delta$. Moreover, for ease of notation, we completely drop the subscript 2.

As a first step, we split the contour into three parts:

3.19 $$\begin{aligned} \gamma =\Gamma _1 \, \dot{+} \, \Gamma _2 \, \dot{+}\, \Gamma _3, \end{aligned}$$

where $\Gamma _1$ is the horizontal part of $\gamma$ with $\Re z \in I_{2\Delta }$, $\Gamma _2$ is the horizontal part of $\gamma$ with $\Re z \notin I_{2\Delta }$ and $\Gamma _3$ consists of the great arc of radius *R* (cf. Fig. 3). We now estimate these three parts separately.

For the first part, $\Gamma _1$, we have that (using the notation  from Lemma A.1)^15^

3.20 $$\begin{aligned}  &   \left| \int _{\Gamma _1} \textrm{e}^{-\textrm{i}t z} \left[ \frac{1}{\mu _j - z - \lambda ^2 m_{\lambda }(z)} - \frac{1}{\mu _j - z - \lambda ^2 m_0(E_0)}\right] \textrm{d}z\right| \nonumber \\  &   \qquad \lesssim \int _{\Gamma _1} \frac{\lambda ^2\, \big (1/t + \lambda + \Delta + \epsilon _0\big )}{|\mu _j - z-\lambda ^2 m_\lambda (z)| \, |\mu _j -z - \lambda ^2 m_0(E_0)| } |\textrm{d}z| \nonumber \\  &   \qquad \lesssim \lambda ^2t \, \big (1/t + \lambda + \Delta + \epsilon _0\big ), \end{aligned}$$

uniformly in $\mu _j \in I_\Delta$. To go to the second line, we used that $|m_\lambda (z) - m_0(E_0)| \lesssim 1/t + \lambda + \Delta + \epsilon _0$. This follows by adding and subtracting $m_\lambda (E_0)$ and using $|m_\lambda (z) - m_\lambda (E_0)| \lesssim \Delta +1/t$ (using $|m_\lambda '(z)| \lesssim 1$ for $\Re z \in I_{2 \Delta }$; cf. the last estimate in (A.2) from Lemma A.1) and $|m_\lambda (E_0) - m_0(E_0)| \lesssim \lambda + \epsilon _0$ (using that (A.7) holds down to the real line by combining it with (A.8)). For the final bound, we employed a Schwarz inequality for the integral and estimated the resulting integrals

$$ \int _{\Gamma _1} \frac{|\textrm{d}z|}{ |\mu _j - z-\lambda ^2 m_\lambda (z)|^2} {\lesssim } (1 + \lambda ^2) \, t {\lesssim } t\quad \text {and} \quad \int _{\Gamma _1} \frac{|\textrm{d}z|}{ |\mu _j - z-\lambda ^2 m_0(E_0)|^2} {\lesssim } t\,, $$

by a change of variables $z \rightarrow z + \lambda ^2 m_\lambda (z)$ using that $|m_\lambda '(z)| \lesssim 1$ for $z \in \Gamma _1$ by means of (A.2) together with $|\Im [z+\lambda ^2 m_\lambda (z)] |\ge t^{-1}$, and $|m_0(E_0)| \lesssim 1$ together with $|\Im [z + \lambda ^2m_0(E_0)] | \ge t^{-1}$, respectively.

We now turn to the second part, i.e. the integral similar to the left-hand side of  (3.20) but on the contour $\Gamma _2$. By means of $|m_0(E_0)| \lesssim 1$ and $|m_\lambda (z)| \le \lambda ^{-1}$ (see the first estimate in (A.1)) we bound $|m_\lambda (z) - m_0(E_0)| \lesssim \lambda ^{-1}$. Using $|m_0(E_0)| \lesssim 1$ and $|m_\lambda (z)| \le \lambda ^{-1}$ again, together with $\textrm{dist}(\mu _j, \Gamma _2) \gtrsim \Delta$, we find this second part to be bounded by (a constant times)

3.21 $$\begin{aligned} \int _{\Gamma _2} \frac{\lambda }{|\mu _j - z|^2} |\textrm{d}z| \, \left( 1 + \frac{\lambda }{\Delta }\right) \lesssim \frac{\lambda }{\Delta } \, \left( 1 + \frac{\lambda }{\Delta }\right) , \end{aligned}$$

again uniformly in $\mu _j \in I_\Delta$.

Finally, we estimate the third part. By the exact same reasoning as for $\Gamma _2$, we arrive at the bound (3.21) with $\Delta$ replaced by *R* and $\Gamma _2$ replaced by $\Gamma _3$. Hence, using that the radius *R* of the semicircle is larger than one (see Fig. 3), we find the third part to be bounded by $\mathcal {O}(\lambda /R)$, uniformly in $\mu _j \in I_\Delta$.

Combining this with the error terms in (3.20) and (3.21), this concludes the proof.

#### Proof of (3.8b)

Recall that $\widetilde{\mathcal {F}}_{\textrm{sing}}$ denotes the singular term defined in (3.8b). To carry out the analog of the approximation (3.9), we observe that the resolvent identity for $H_0$ implies

3.22 $$\begin{aligned} \frac{z_{1,\lambda }-z_{2,\lambda }}{z_1-z_2}M_0(z_{1,\lambda })M_0(z_{2,\lambda })  &   = \frac{M_0(z_{1,\lambda })-M_0(z_{2,\lambda })}{z_1-z_2} \nonumber \\    &   = \frac{1}{\pi }\int _\textbf{R}\frac{\Im M_0(x_\lambda )}{(x-z_1)(x-z_2)}\textrm{d}x, \end{aligned}$$

where we introduce the notation $z_{1,\lambda }:= z_1+\lambda ^2\overline{m_0(E_0)}$, $z_{2,\lambda }:= z_2+\lambda ^2m_0(E_0)$, and $x_\lambda := x + \lambda ^2m_0(E_0)$. Note that $x_\lambda$ is a complex number with $\Im x_\lambda = \lambda ^2 \Im m_0(E_0) > 0$. Here, the second equality follows from the contour representation of the resolvent $M_0$ of $H_0$, namely

3.23 $$\begin{aligned} M_0(z) = \frac{1}{\pi }\int _\textbf{R}\frac{\Im M_0(x+\textrm{i}\eta )}{x+\textrm{i}\eta -z}\textrm{d}x, \quad \Im z>\eta >0. \end{aligned}$$

On the other hand, subtracting two instances of the MDE (3.1) yields3.24where we used the matrix-valued analog of the Stieltjes representation for $M_\lambda (z)$ (cf. [3, Prop. 2.1]),

3.25 $$\begin{aligned} M_\lambda (z)=\frac{1}{\pi }\int _\textbf{R}\frac{\Im M_\lambda (x)}{x-z}\textrm{d}x, \quad z \in \textbf{C}\setminus \textbf{R}. \end{aligned}$$

In particular, identities (3.22) and (3.24) suggest that the appropriate approximation for the factor  in the denominator of (3.8b) is $(z_1-z_2)(z_{1,\lambda }-z_{2,\lambda })^{-1}$. Indeed, we prove that the following estimate holds.

##### Lemma 3.4

Under the Assumption 2.2 and 2.3, the singular term $\widetilde{\mathcal {F}}_{\textrm{sing}}$ defined in (3.8b) satisfies

3.26 $$\begin{aligned} \bigl |\widetilde{\mathcal {F}}_{\textrm{sing}}- \mathcal {F}_{\textrm{sing}}\bigr |\lesssim \mathcal {E}_{\textrm{sing}}:= (\Delta +\epsilon _0)(1+\lambda ^2t)+ \lambda \bigl (1+\lambda ^2t+\Delta ^{-1}\log t\bigr )^2,\nonumber \\ \end{aligned}$$

where the quantity $\mathcal {F}_{\textrm{sing}}$ is given by3.27

We defer the proof of Lemma 3.4 to the end of the current Sect. 3.2.2, and proceed to analyze the right-hand side of (3.27).

Applying the identity (3.22) to both traces in the integrand of (3.27), we obtain the expression3.28where the function $F_{\lambda ,t}(x,y)$ is defined as

3.29 $$\begin{aligned} F_{\lambda ,t}(x,y):=\frac{\lambda ^2}{(2\pi \textrm{i})^2\pi ^2}\oint _{\gamma _1}\oint _{\gamma _2}\frac{ \textrm{e}^{\textrm{i}t (z_1 - z_2)} \, (z_1-z_2)}{(x-z_1)(x-z_2)(y-z_1)(y-z_2)(z_{1,\lambda }-z_{2,\lambda })}\textrm{d}z_2\textrm{d}z_1.\nonumber \\ \end{aligned}$$

Recall the contours $\gamma _1$ and $\gamma _2$ from Fig. 3, and that $z_{1,\lambda }-z_{2,\lambda } = z_1-z_2-2\textrm{i}\alpha \lambda ^2$. Evaluating the contour integration over $\gamma _2$ in (3.29) yields

3.30 $$\begin{aligned} \begin{aligned} F_{\lambda ,t}(x,y) = \frac{\lambda ^2}{\pi ^2}\frac{1}{2\pi \textrm{i}}\oint _{\gamma _1}&\biggl (\frac{-\textrm{e}^{\textrm{i}t(z_1-x)}\chi (x)}{(x-y)(y-z_1)(x_\lambda -z_{1,\lambda })} +\frac{\textrm{e}^{\textrm{i}t(z_1-y)}\chi (y)}{(x-y)(x-z_1)(y_\lambda -z_{1,\lambda })}\\&+\frac{2\textrm{i}\alpha \lambda ^2\textrm{e}^{-2\alpha \lambda ^2t}}{(x-z_1)(y-z_1)(x_\lambda -z_{1,\lambda })(y_\lambda -z_{1,\lambda })}\biggr )\textrm{d}z_1 - F_{\lambda ,t}^{\textrm{out}}(x,y), \end{aligned}\nonumber \\ \end{aligned}$$

where we define $\chi (z):=\mathbbm {1}_{\Omega _2}(z)$, and $\Omega _2$ is the compact connected component of $\textbf{C}\backslash \gamma _2$, and the function $F_{\lambda ,t}^{\textrm{out}}(x,y)$ is defined as

3.31 $$\begin{aligned} F_{\lambda ,t}^{\textrm{out}}(x,y):= \frac{\lambda ^2}{\pi ^2}\frac{1}{2\pi \textrm{i}}\oint _{\gamma _1} \frac{2\textrm{i}\alpha \lambda ^2\textrm{e}^{-2\alpha \lambda ^2t} \bigl (1-\chi (z_1-2\textrm{i}\alpha \lambda ^2)\bigr )}{(x-z_1) (y-z_1)(x_\lambda -z_{1,\lambda }) (y_\lambda -z_{1,\lambda })}\textrm{d}z_1.\nonumber \\ \end{aligned}$$

We proceed to show that $F_{\lambda ,t}^{\textrm{out}}(x,y)$ contributes at most an $\mathcal {O}(\lambda ^4(1+|\log \lambda |^2+ (\lambda ^2t)^2))$ error to the right-hand side of (3.28). Let $\gamma _{1,c}$ denote the set (see Fig. 3)

3.32 $$\begin{aligned} \gamma _{1,c}:= \{z_1\in \gamma _1: z_1-2\textrm{i}\alpha \lambda ^2 \notin \Omega _2\}. \end{aligned}$$

Then the contribution of $F_{\lambda ,t}^{\textrm{out}}(x,y)$ to the integral in (3.28) is given by3.33Note that by choosing the radii of the contours $R\gtrsim 1$ large enough, we can assume that

3.34 $$\begin{aligned} \textrm{dist}(\sigma (H_0),\gamma _{1,c})\gtrsim R. \end{aligned}$$

Using the spectral decomposition of $H_0$ from (3.11), the fact that $\int _\textbf{R}\Im [(\mu _j-x_\lambda )]\textrm{d}x = \pi$, and Assumption 2.3, we conclude that3.35Furthermore, the spectral decomposition for $H_0$ implies that for all *x* with $\textrm{dist}(x,\sigma (H_0)) \gtrsim 1$,3.36Let $x_\pm$ denote the intersections of the contour $\gamma _1$ with $\textbf{R}$ (see Fig. 3), and define $\mathbb {D}$ to be a union of two small disks of radius $\varepsilon$ around $x_\pm$, $\mathbb {D}:= \{z\in \textbf{C}: \min \{|z-x_-|,|z-x_+|\} \le \varepsilon \}$, for a sufficiently small constant $\varepsilon \sim 1$ . Applying the Cauchy-Schwarz inequality to the $z_1$ integration in (3.33), and using the estimates (3.34)-(3.36) to bound the contribution coming from the outside of $\mathbb {D}$, and applying the estimates (3.34) and (3.36) for all $x,y\in \textbf{R}\cap \mathbb {D}$, we obtain

3.37 $$\begin{aligned} \bigl |\mathcal {E}_{\gamma _{1,c}} \bigr |\lesssim \lambda ^4 \int _{\gamma _{1,c}\cap \mathbb {D}} \biggl |\frac{\lambda ^2}{R^2}\int _{\textbf{R}\cap \mathbb {D}}\frac{\textrm{d}x}{|x-z_1||x_\lambda -z_{1,\lambda }|} \biggr |^2 |\textrm{d}z_1| + \mathcal {O}\bigl ( R^{-3}\lambda ^4\bigr ).\nonumber \\ \end{aligned}$$

For $z_1$ on the horizontal linear segment of $\gamma _{1,c}\cap \mathbb {D}$, we use that $\Im z_1 = -1/t$ to obtain

3.38 $$\begin{aligned} \frac{\lambda ^2}{R^2}\int _{\textbf{R}\cap \mathbb {D}}\frac{\textrm{d}x}{|x-z_1||x_\lambda -z_{1,\lambda }|} \lesssim \frac{1 + \lambda ^2 t}{R^2}, \end{aligned}$$

On the other hand, for $z_1$ lying on the circular arc parts of $\gamma _{1,c}\cap \mathbb {D}$, we compute

3.39 $$\begin{aligned} \frac{\lambda ^2}{R^2}\int _{\textbf{R}\cap \mathbb {D}}\frac{\textrm{d}x}{|x-z_1||x_\lambda -z_{1,\lambda }|} \lesssim \frac{\lambda ^2}{R^2}\frac{ |\log \lambda ^2| + \bigl |\log |\eta _1|\bigr |+\bigl |\log |\eta _1 - 2\alpha \lambda ^2|\bigr |}{|\eta _1| + \lambda ^2},\nonumber \\ \end{aligned}$$

where $\eta _1:= \Im z_1$. Squaring the estimates (3.38) and (3.39) and integrating them over the respective parts of $\gamma _{1,c}\cap \mathbb {D}$, we conclude from (3.37) that

3.40 $$\begin{aligned} \bigl |\mathcal {E}_{\gamma _{1,c}} \bigr |\lesssim R^{-3}\lambda ^4 \bigl (1+ (\lambda ^2t)^2+|\log \lambda |^2\bigr ). \end{aligned}$$

Next, using residue calculus, we compute the first term on the right-hand side of (3.30), i.e., the contour integral over $\gamma _1$, to obtain

3.41 $$\begin{aligned} F_{\lambda ,t}(x,y)= &   ~\frac{K_{\lambda ,t}(x-y)}{\alpha \pi }-F_{\lambda ,t}^{\textrm{out}}(x,y) + \frac{K_{\lambda ,t}(x-y)}{\alpha \pi }\bigl (\chi (x)\chi (y)-1\bigr ) \nonumber \\  &   \quad + \frac{1}{2\pi ^2}\frac{2\lambda ^2\textrm{e}^{-2\alpha \lambda ^2t}\bigl (\chi (x)-\chi (y)\bigr )^2}{|x-y|^2 + (2\alpha \lambda ^2)^2}\nonumber \\  &   \quad + \frac{1}{\pi ^2}\frac{2\textrm{i}\alpha \lambda ^4\textrm{e}^{-2\alpha \lambda ^2t}}{|x-y|^2+(2\alpha \lambda ^2)^2}\frac{\chi (x)-\chi (y)}{x-y}, \end{aligned}$$

where $K_{\lambda ,t}$ is the kernel defined in (2.10). As we have proved above, the contribution of $F_{\lambda ,t}^{\textrm{out}}(x,y)$ to the integral in (3.28) admits the bound (3.40). Similarly, using the estimates (3.35) and (3.36), it is straightforward to check that the third and fourth terms on the right-hand side of (3.41) contribute at most $\mathcal {O}(\lambda ^4)$ to the right-hand side of (3.28), while the last term contributes at most $\mathcal {O}(\lambda ^2)$. Therefore,

3.42 $$\begin{aligned} \mathcal {F}_{\textrm{sing}}= \langle A \rangle _{\widetilde{P}_{\lambda ,t}}\big (1+ \mathcal {O}(\epsilon _0 + \Delta + \lambda ^2/\Delta )\big ) + \mathcal {O}(\lambda ^2), \end{aligned}$$

where we used the estimate $\pi \alpha = \pi ^2\rho _0(E_0) = N^{-1}r\big (1+ \mathcal {O}(\epsilon _0 + \Delta + \lambda ^2/\Delta )\big )$ that follows from Lemma A.2 for *r* defined in (2.22), and performed a change of variables $x \rightarrow x - \lambda ^2\Re m_0(E_0)$ and $y \rightarrow y - \lambda ^2\Re m_0(E_0)$. We note that the $N^{-1}$ prefactor results from the different normalization of the trace in (2.22) and (A.14). Furthermore, Proposition 3.6 below implies that under the Assumptions 2.2 and 2.3, $|\langle A \rangle _{\widetilde{P}_{\lambda ,t}} |\lesssim 1$. Since the proof of Proposition 3.6 is independent of the statement of (3.8b), this concludes the proof of (3.8b).

We proceed to prove Lemma 3.4.

##### Proof of Lemma 3.4

Define the sequences of overlaps $\mathfrak {a}_j:= \langle \varvec{u}_j, A\varvec{u}_j\rangle$, and $\mathfrak {p}_k:= \langle \varvec{u}_k, P\varvec{u}_k\rangle$ where we recall from (3.11) that $\varvec{u}_j$’s are the eigenvectors of $H_0$. Observe that the Assumption 2.3 implies that

3.43 $$\begin{aligned} \Vert \mathfrak {a}\Vert _\infty \lesssim 1, \quad \mathfrak {p}_k \ge 0 \quad \text { and }\quad \Vert \mathfrak {p}\Vert _1 = 1. \end{aligned}$$

Then, using the spectral decomposition (3.11) of $H_0$ and the identity (3.24), we rewrite $\widetilde{\mathcal {F}}_{\textrm{sing}}$ in the following form,

3.44 $$\begin{aligned} \widetilde{\mathcal {F}}_{\textrm{sing}}= \sum _{j,k}\frac{\mathfrak {a}_j\mathfrak {p}_k}{N}\frac{1}{\pi }\int _\textbf{R}\Im \widetilde{\nu }_j(x)\lambda ^2\bigl |\widetilde{\omega }_k(x)\bigr |^2 \textrm{d}x, \end{aligned}$$

where $\widetilde{\nu }_j(z):= (\mu _j-z-\lambda ^2m_\lambda (z))^{-1}$ for $z \in \textbf{C}$, and the functions $\widetilde{\omega }_k(x)$ are defined by the improper integrals^16^

3.45 $$\begin{aligned} \widetilde{\omega }_k(x):= \frac{1}{2\pi \textrm{i}}\oint \limits _{\gamma _2} \frac{\textrm{e}^{\textrm{i}t z}\widetilde{\nu }_k(z)}{x-z} \textrm{d}z, \quad x \in \textbf{R}. \end{aligned}$$

Here we adhere to the convention $m_\lambda (x):= \lim _{\eta \rightarrow +0} m_\lambda (x+\textrm{i}\eta )$.

The key estimates for proving (3.26) are collected in the following Lemma that we prove at the end of the subsection.

##### Lemma 3.5

(Second replacement lemma). Define the functions

3.46 $$\begin{aligned} \omega _k(x):= \frac{1}{2\pi \textrm{i}}\oint _{\gamma _2} \frac{\textrm{e}^{-\textrm{i}t z}}{x-z} \frac{1}{\mu _k - z_\lambda } \textrm{d}z, \quad k\in [N], \end{aligned}$$

where we denote $z_\lambda := z + \lambda ^2m_0(E_0)$. Then under the Assumptions 2.2 and 2.3, the estimates

3.47 $$\begin{aligned}  &   \bigl |\omega _k(x)-\widetilde{\omega }_k(x) \bigr |\lesssim \frac{(\Delta +\epsilon _0)(1+\lambda ^2t)}{|\mu _k-x_\lambda |} + 1 + \lambda ^2t+\Delta ^{-1}\log t, \quad x\in I_{3\Delta },\nonumber \\ \end{aligned}$$

3.48 $$\begin{aligned}  &   \bigl |\omega _k(x)\bigr |+ \bigl |\widetilde{\omega }_k(x) \bigr |\lesssim \frac{1}{|\mu _k-x_\lambda |} + 1 + \lambda ^2t+\Delta ^{-1}\log t,\quad x\in I_{3\Delta }, \end{aligned}$$

3.49 $$\begin{aligned}  &   \bigl |\omega _k(x)\bigr |+ \bigl |\widetilde{\omega }_k(x) \bigr |\lesssim \Delta ^{-1}\log t+R^{-1}, \quad x\in [-\tfrac{1}{2}R,\tfrac{1}{2}R]\backslash I_{3\Delta }, \end{aligned}$$

hold for all *k* with $\mu _k \in I_\Delta$.

Observe that applying the identities (3.22), (3.24), and the spectral decomposition of $H_0$ to (3.28) yields

3.50 $$\begin{aligned} \widetilde{\mathcal {F}}_{\textrm{sing}}- \mathcal {F}_{\textrm{sing}}= \mathcal {E}_{\textrm{sing},1} + \mathcal {E}_{\textrm{sing},2}, \end{aligned}$$

where, recalling $x_\lambda := x-\lambda ^2m_0(E_0)$, the quantities $\mathcal {E}_{\textrm{sing},1}$ and $\mathcal {E}_{\textrm{sing},2}$ are defined as

3.51 $$\begin{aligned}  &   \mathcal {E}_{\textrm{sing},1}:= \sum _{j,k}\frac{\mathfrak {a}_j\mathfrak {p}_k}{N}\frac{1}{\pi }\int _\textbf{R}\Im \bigl [\widetilde{\nu }_j(x)- (\mu _j-x_\lambda )^{-1}\bigr ] \lambda ^2\bigl |\widetilde{\omega }_k(x)\bigr |^2\textrm{d}x. \end{aligned}$$

3.52 $$\begin{aligned}  &   \mathcal {E}_{\textrm{sing},2}:= \sum _{j,k}\frac{\mathfrak {a}_j\mathfrak {p}_k}{N}\frac{1}{\pi }\int _\textbf{R}\Im \bigl [(\mu _j-x_\lambda )^{-1}\bigr ]\lambda ^2 \biggl (\bigl |\widetilde{\omega }_k(x)\bigr |^2- \bigl |\omega _k(x)\bigr |^2\biggr )\textrm{d}x.\nonumber \\ \end{aligned}$$

First, we estimate the quantity $\mathcal {E}_{\textrm{sing},1}$ defined in (3.51). In the regime $x\in I_{3\Delta }$, the bounds in (A.2) imply that

3.53 $$\begin{aligned} \bigl |\widetilde{\nu }_j(x)-(\mu _j-x_\lambda )^{-1}\bigr |\lesssim (\Delta +\epsilon _0) \frac{\lambda ^2}{|\mu _j-x_\lambda |^2}, \quad x \in I_{3\Delta }. \end{aligned}$$

On the other hand, it is straightforward to check that the regime $|x| \ge \frac{1}{2}R$ contributes at most $\mathcal {O}(\lambda ^2 R^{-2})$ to the integral on the right-hand side of (3.51). We note that the logarithmic singularity resulting from the contour $\gamma _2$ intersecting the real line is removed by the *x* integration.

Therefore, estimates (3.43), (3.48), (3.49), and (3.53) imply that

3.54 $$\begin{aligned} \begin{aligned} \bigl |\mathcal {E}_{\textrm{sing},1} \bigr |\lesssim&~ \sum _{j,k}\frac{|\mathfrak {a}_j|\mathfrak {p}_k}{N}\int _{I_{3\Delta }}\frac{\lambda ^2(\Delta +\epsilon _0)}{|\mu _j-x_\lambda |^2} \frac{\lambda ^2}{|\mu _k-x_\lambda |^2}\textrm{d}x\\&+\lambda ^2(1 + \lambda ^2t+\Delta ^{-1}\log t)^2\sum _{j}\frac{1}{N} \int _{\textbf{R}}\Im \bigl [\widetilde{\nu }_j(x) + (\mu _j-x_\lambda )^{-1}\bigr ] \textrm{d}x \\ \lesssim&~\sum _{j,k}\frac{|\mathfrak {a}_j|\mathfrak {p}_k}{N}\int _{\textbf{R}} \frac{\lambda ^2(\Delta +\epsilon _0)}{|\mu _j-x_\lambda |^2} \frac{\lambda ^2}{|\mu _k-x_\lambda |^2}\textrm{d}x + \lambda ^2\bigl (1+\lambda ^2 t + \Delta ^{-1}\log t\bigr )^2, \end{aligned}\nonumber \\ \end{aligned}$$

where in the first step we used the bound $|\Im [\widetilde{\nu }_j(x) - (\mu _j-x_\lambda )^{-1}]| \le \Im [\widetilde{\nu }_j(x) + (\mu _j-x_\lambda )^{-1}]$ to estimate the contribution coming from the second term on the right-hand side of (3.48) and the regime $x \in [-\frac{1}{2}R, \frac{1}{2}R] \backslash I_{3\Delta }$; and in the second step we used that $\int _\textbf{R}\Im [(\mu _j-x_\lambda )^{-1}] \textrm{d}x = \pi$ and $\int _\textbf{R}\frac{1}{N}\sum _j \Im \widetilde{\nu }_j(x)\textrm{d}x = \int _R \Im m_\lambda (x)\textrm{d}x = \pi$ (see, e.g., Proposition 2.1 and Eq. (2.9) in [3]). Computing the integral in the second term on the right-hand side of (3.54) explicitly, and using the spectral decomposition of $H_0$, we deduce from the admissibility of $E_0$ that3.55Here we employed (3.43) and the estimate $\langle \mathfrak {a}, X \mathfrak {p}\rangle \lesssim \Vert \mathfrak {a}\Vert _\infty \Vert \mathfrak {p}\Vert _1 \sup \limits _{k\in \textrm{supp}(\mathfrak {p})} \sum _j |X_{jk}|$. Hence, we conclude that

3.56 $$\begin{aligned} \bigl |\mathcal {E}_{\textrm{sing},1} \bigr |\lesssim \Delta +\epsilon _0+ \lambda ^2\bigl (1+\lambda ^2t+\Delta ^{-1}\log t\bigr )^2. \end{aligned}$$

We proceed to estimate the quantity $\mathcal {E}_{\textrm{sing},2}$ defined in (3.52). We note again that the contribution of the regime $|x| \ge \frac{1}{2}R$ to the integral on the right-hand side of (3.52) is bounded by $\mathcal {O}(\lambda ^2R^{-2})$. Therefore, combining the estimates (3.47), (3.48), and (3.49) yields the bound

3.57 $$\begin{aligned} \begin{aligned} \bigl |\mathcal {E}_{\textrm{sing},2}\bigr |\lesssim (\Delta +\epsilon _0)(1+\lambda ^2t) +\lambda \bigl (1+\lambda ^2t+\Delta ^{-1}\log t\bigr )^2, \end{aligned} \end{aligned}$$

obtained similarly to (3.54) and (3.56). Together with (3.50), the bounds (3.56) and (3.57) conclude the proof of (3.26). $\square$

It remains to prove Lemma 3.5.

##### Proof of Lemma 3.5

Throughout the proof we assume that $k\in [N]$ satisfies $\mu _k \in I_{\Delta }$, and $x\in \textbf{R}$ satisfies $|x| \le \frac{1}{2}R$. We introduce the auxiliary quantities

3.58 $$\begin{aligned} \check{\nu }_k(z):= \frac{1}{\mu _k - z -\lambda ^2m_\lambda (\mu _k)}, \quad \check{\omega }_k(x):= \frac{1}{2\pi \textrm{i}}\oint _{\gamma _2}\frac{\textrm{e}^{-\textrm{i}tz}}{x-z}\check{\nu }_k(z)\textrm{d}z. \end{aligned}$$

An explicit computation using the residue calculus reveals that

3.59 $$\begin{aligned} \check{\omega }_k(x) = \frac{\textrm{e}^{-\textrm{i}t x } - \textrm{e}^{-\textrm{i}t (\mu _k - \lambda ^2m_\lambda (\mu _k))}}{\mu _k -x - \lambda ^2m_\lambda (\mu _k)}, \quad \omega _k(x) = \frac{\textrm{e}^{-\textrm{i}t x } - \textrm{e}^{-\textrm{i}t (\mu _k - \lambda ^2 m_0(E_0))}}{\mu _k -x_\lambda }.\nonumber \\ \end{aligned}$$

Furthermore, using the bound in (A.2), we obtain

3.60 $$\begin{aligned} \bigl |\omega _k(x) - \check{\omega }_k(x) \bigr |\lesssim \biggl (\frac{1}{|\mu _k-x_\lambda |^2} + \frac{t}{|\mu _k-x_\lambda |}\biggr )\lambda ^2|\mu _k - E_0| \lesssim \frac{\Delta (1+\lambda ^2 t)}{|\mu _k-x_\lambda |},\nonumber \\ \end{aligned}$$

where we additionally applied the estimate

3.61 $$\begin{aligned} \bigl |y+\mathcal {O}(\eta )\bigr | + \eta \sim |y| + \eta , \quad y\in \textbf{R}, \eta >0. \end{aligned}$$

We decompose the contour $\gamma _2 = \Gamma _1\,\dot{+}\,\Gamma _2 \,\dot{+}\,\Gamma _3$ according to (3.19). It is straightforward to check that for $\nu ^\#(z)$ denoting one of $\widetilde{\nu }_k(z)$, $\check{\nu }_k(z)$ or $(\mu _k - z_\lambda )^{-1}$,

3.62 $$\begin{aligned} \biggl |\int _{\Gamma _2\dot{+}\Gamma _3} \frac{\textrm{e}^{-\textrm{i}tz}}{x-z}\nu ^\#_k(z)\textrm{d}z \biggr |\lesssim \frac{\log t}{\Delta } + \frac{1}{R}, \end{aligned}$$

where we used that $\Im z = t^{-1}$ for all $z\in \Gamma _1$. Therefore, rewriting the left-hand sides of (3.47)-(3.49) using the integral definitions (3.45) and (3.46), it suffices to estimate the contributions coming from the segment $\Gamma _1\subset \gamma _2$.

Using (3.61) and (A.2), we deduce that for all $z\in \Gamma _1$, defined in (3.19),

3.63 $$\begin{aligned} \bigl |\check{\nu }_k(z) - \widetilde{\nu }_k(z) \bigr |\lesssim \frac{\lambda ^2}{|\mu _k-z_\lambda |}. \end{aligned}$$

Integrating the bound (3.63) then yields

3.64 $$\begin{aligned} \biggl |\int _{\Gamma _1} \frac{\textrm{e}^{-\textrm{i}tz}}{x-z}\bigl [\widetilde{\nu }_k(z)-\check{\nu }_k(z)\bigr ]\textrm{d}z \biggr |\lesssim \bigl ( 1 + \lambda ^2t \bigr )\mathbbm {1}_{x\in I_{3\Delta }} + \bigl (\Delta ^{-1}\log t\bigr ) \mathbbm {1}_{x\notin I_{3\Delta }},\nonumber \\ \end{aligned}$$

which, together with (3.60), (3.62) immediately implies (3.47) after writing $\widetilde{\omega }_k - \omega _k = (\widetilde{\omega }_k-\check{\omega }_k) + (\check{\omega }_k-\omega _k)$.

On the other hand, noting that $|\omega _k(x)|+ |\check{\omega }_k(x)| \lesssim |\mu _k-x_\lambda |^{-1}$ by (3.59), and combining the estimates (3.60) and (3.62) yields (3.48) and (3.49). This concludes the proof of Lemma 3.5.

### Step (iii): Limiting Behavior of the Singular Term and Proof of Theorem 2.4 (b)

We organize the result of approximating $\langle A \rangle _{\widetilde{P}_{\lambda ,t}}$ in the following proposition.

#### Proposition 3.6

Under the assumptions of Theorem 2.4, and with $\widetilde{P}_\lambda$ defined as in (2.12), we have that, for any fixed $T \in (0,\infty )$ and recalling $\alpha = \pi \rho _0(E_0)$

$$\begin{aligned} \limsup _{\Delta \rightarrow 0}\limsup _{\begin{array}{c} t\rightarrow \infty \\ \lambda \rightarrow 0\\ \lambda ^2 t=T \end{array}}\limsup _{N\rightarrow \infty } \bigl |\langle A \rangle _{\widetilde{P}_{\lambda ,t}} - (1- \textrm{e}^{-2\alpha \lambda ^2t})\langle A \rangle _{\widetilde{P}_\lambda } \bigr |\lesssim T\textrm{e}^{-2\alpha T}. \end{aligned}$$

Given Proposition 3.6, Theorem 2.4 (b) immediately follows. $\square$

#### Proof of Proposition 3.6

First, we observe that representing $\Im M_\lambda$ in spectral decomposition of $H_0$, the quantity $\langle A \rangle _{\widetilde{P}_{\lambda ,t}}$ with $P_{\lambda , t}$ defined in (2.9), can be rewritten in the from

3.65 $$\begin{aligned} \langle A \rangle _{\widetilde{P}_\lambda ,t} = \frac{1}{r}\sum _{j,k}\mathfrak {a}_j\mathfrak {p}_k\int _{\textbf{R}} \phi _{\alpha \lambda ^2}(x-\mu _j) \bigl (K_{\lambda ,t} * \phi _{\alpha \lambda ^2}\bigr )(x-\mu _k) \textrm{d}x, \end{aligned}$$

where $r=\int _\textbf{R}\textrm{Tr}[\Im M_0(x + \textrm{i}\alpha \lambda ^2)]\langle \Im M_0(x + \textrm{i}\alpha \lambda ^2)\rangle _P\textrm{d}x > 0$ has already been introduced in Remark 2.8 (ii), and we denoted $\phi _{\eta }:= \Im [(x-\textrm{i}\eta )^{-1}]$. Recall that $\mu _j, \varvec{u}_j$ are the eigenvalues and the respective eigenvectors of $H_0$, and $\mathfrak {a}_j:= \langle \varvec{u}_j, A\varvec{u}_j\rangle$, $\mathfrak {p}_j:= \langle \varvec{u}_j, P\varvec{u}_j\rangle$. Applying the Parseval-Plancherel identity to the right-hand side of (3.65) yields

3.66 $$\begin{aligned}  &   \langle A \rangle _{\widetilde{P}_{\lambda ,t}} = \frac{1}{r}\sum _{j,k}\mathfrak {a}_j\mathfrak {p}_k \Phi _{\lambda ,t}(\mu _j-\mu _k), \quad \nonumber \\    &   \Phi _{\lambda ,t}(u):= \frac{\pi ^2}{(2\pi )^{1/2}}\int _\textbf{R}\textrm{e}^{-2\alpha \lambda ^2|q|-\textrm{i}u q}\, \widehat{K_{\lambda ,t}}(q)\textrm{d}q, \end{aligned}$$

t $$\widehat{\phi _\eta }(q) = (\frac{\pi }{2})^{1/2}\textrm{e}^{-\eta |q|},\, \eta >0$$ (recall Footnote 5).

A direct computation starting with (2.10) reveals that

3.67 $$\begin{aligned} \widehat{K_{\lambda ,t}}(q) = {\left\{ \begin{array}{ll} (2\pi )^{-1/2}\bigl (1 - \textrm{e}^{-2\alpha \lambda ^2 (t-|q|)} \bigr ) \quad & \text {for} \quad |p| \le t, \\ 0 \quad & \text {for} \quad |p| > t, \end{array}\right. } \end{aligned}$$

and implies that $\Phi _{\lambda ,t}(u)$ admits the explicit expression

3.68 $$\begin{aligned} \Phi _{\lambda ,t}(u) = \bigl (1-\textrm{e}^{-2\alpha \lambda ^2 t}\bigr ) \pi \phi _{2\alpha \lambda ^2}(u)+ \mathfrak {R}_{\lambda ,t}(u), \end{aligned}$$

where the function $\mathfrak {R}_{\lambda , t}(u)$ is defined by

3.69 $$\begin{aligned} \mathfrak {R}_{\lambda ,t}(u):= \pi \textrm{e}^{-2\alpha \lambda ^2 t}\phi _{2\alpha \lambda ^2}(u)\biggl (1-\cos (tu)-2\alpha \lambda ^2 t \frac{\sin (tu)}{tu}\biggr ). \end{aligned}$$

Observe that the contribution of the first term on the right-hand side of (3.68) to $\langle A \rangle _{\widetilde{P}_{\lambda ,t}}$ is given by $(1-\textrm{e}^{-2\lambda ^2\alpha t})\langle A \rangle _{\widetilde{P}_\lambda }$, since

3.70 $$\begin{aligned} \langle A \rangle _{\widetilde{P}_\lambda } = \frac{1}{r}\sum _{j,k}\mathfrak {a}_j\mathfrak {p}_k\int _\textbf{R}\phi _{\alpha \lambda ^2}(x-\mu _j) \phi _{\alpha \lambda ^2}(x-\mu _k)\textrm{d}x. \end{aligned}$$

Here we used the definition of the state $\widetilde{P}_\lambda$ in (2.12), and the Parseval-Plancherel identity.

The key observation is that the contribution of the remaining $\mathfrak {R}_{\lambda ,t}(\mu _j-\mu _k)$ term

3.71 $$\begin{aligned} \mathcal {R}:= \sum _{j,k}\frac{\mathfrak {a}_j\mathfrak {p}_k}{r} \mathfrak {R}_{\lambda ,t}(\mu _j-\mu _k) \end{aligned}$$

in (3.68) to $\langle A \rangle _{\widetilde{P}_{\lambda ,t}}$ admits the bound^17^

3.72 $$\begin{aligned} \big |\mathcal {R}\big |\le \sup _{k\in \textrm{supp}(\mathfrak {p})}\frac{1}{r}\sum _{j} \bigl |\mathfrak {R}_{\lambda ,t}(\mu _j-\mu _k) \bigr |\cdot \Vert \mathfrak {a}\Vert _\infty \Vert \mathfrak {p}\Vert _1. \end{aligned}$$

Observe that there exists a constant $C>0$ such that for any $\xi >0$ and $t>0$, we have that $\phi _{ \xi }(u) (1-\cos (tu)) \le C\xi t \phi _{1/t}(u)$ for all $u \in \textbf{R}$. This follows immediately from the fact that the function $s \mapsto (s^2+1)(1-\cos s)/s^2$ is uniformly bounded on $\textbf{R}$. Therefore, the function $\mathfrak {R}$ admits the bound

3.73 $$\begin{aligned} |\mathfrak {R}_{\lambda ,t}(u)| \le 2\pi \alpha \lambda ^2 t\,\textrm{e}^{-2\alpha \lambda ^2 t}\biggl (C\phi _{1/t}(u)+\phi _{2\alpha \lambda ^2}(u)\biggr ), \quad u\in \textbf{R}. \end{aligned}$$

Summing the bound (3.73) over $u = \mu _j$ yields3.74Using the localization of the state *P* as in (2.6), the admissibility of $E_0$ in (2.5), and the first line of (A.14) to deduce that $r \sim N(1+ \mathcal {O}(\epsilon _0 + \Delta + \lambda ^2/\Delta ))$, we obtain

3.75 $$\begin{aligned} \sup _{k\in \textrm{supp}(\mathfrak {p})}\frac{1}{r}\sum _{j} \bigl |\mathfrak {R}_{\lambda ,t}(\mu _j-\mu _k) \bigr | \lesssim \lambda ^2 t\,\textrm{e}^{-2\alpha \lambda ^2 t}\bigl (1 + \epsilon _0\bigr )\big (1+\epsilon _0 + \Delta + \lambda ^2/\Delta \big ).\nonumber \\ \end{aligned}$$

This concludes the proof of Proposition 3.6. $\square$

### Relaxation formula: Proof of Corollary 2.7

Estimates (2.18) and (2.19) in items (a) and (b), respectively, follow immediately from Theorem 2.4.

To prove (2.20) in item (c), observe that plugging the estimate (2.16) from the Definition 2.6 of local overlap regularity into (3.71) yields

3.76 $$\begin{aligned} \bigl |\mathcal {R}\bigr |&\lesssim ~ |\mathfrak {A}| \sup \limits _{k\in \textrm{supp}(\mathfrak {p})} \biggl |\frac{1}{N}\sum _{\mu _j\in I_{2\Delta }} \mathfrak {R}_{\lambda ,t}(\mu _j-\mu _k) \biggr |\nonumber \\&\quad + \sup \limits _{k\in \textrm{supp}(\mathfrak {p})} \biggl |\sum _{\mu _j\in I_{2\Delta }} \frac{\mathfrak {a}_j-\mathfrak {A}}{N} \mathfrak {R}_{\lambda ,t}(\mu _j-\mu _k)\biggr |\nonumber \\&\quad + \sup \limits _{k\in \textrm{supp}(\mathfrak {p})} \biggl |\frac{1}{N}\sum _{\mu _j\notin I_{2\Delta }} \mathfrak {a}_j \mathfrak {R}_{\lambda ,t}(\mu _j-\mu _k) \biggr |, \end{aligned}$$

where we used that $|r| \sim N\, (1+ \mathcal {O}(\epsilon _0 + \Delta + \lambda ^2/\Delta ))$ by the first line of (A.14) from Lemma A.2.

Applying the estimates analogous to (3.72) and (3.75) to the second sum on the right-hand side of (3.76), we deduce the bound

3.77 $$\begin{aligned} \sup \limits _{k\in \textrm{supp}(\mathfrak {p})} \biggl |\sum _{\mu _j\in I_{2\Delta }} \frac{\mathfrak {a}_j-\mathfrak {A}}{N} \mathfrak {R}_{\lambda ,t}(\mu _j-\mu _k)\biggr |\lesssim \big | \mathcal {E}_{\textrm{LOR}} \big | \cdot \big (1+\lambda ^2/\Delta \big ) \end{aligned}$$

Note that by (3.43) and the uniform bound

$$ |\mathfrak {R}_{\lambda ,t}(u)| \lesssim \frac{\lambda ^2}{\Delta ^2}, \quad \text{ for } \quad |u| \gtrsim \Delta $$

following from (3.73), the tail sum, i.e., the second line of (3.76), admits the estimate

3.78 $$\begin{aligned} \sup \limits _{k\in \textrm{supp}(\mathfrak {p})} \biggl |\frac{1}{N}\sum _{\mu _j\notin I_{2\Delta }} \mathfrak {a}_j \mathfrak {R}_{\lambda ,t}(\mu _j-\mu _k) \biggr |\lesssim \frac{\lambda ^2}{ \Delta ^2}. \end{aligned}$$

Therefore, it remains to estimate the first term on the right-hand side of (3.76). Since the function $\mathfrak {R}_{\lambda ,t}(u)$ is holomorphic in *u* for $|\Im u| \le \alpha \lambda ^2$, we obtain the following series of estimates,3.79where the contour $\gamma$ is defined to be a rectangle of height $2\eta _0$ and width 4*C* centered at $E_0$, and the constant $C\sim 1$ is chosen in such a way that $\sigma (H_0) \subset [E_0-C,E_0+C]$. Here, in the first step, we used residue calculus and an estimate analogous to (3.78) to extend the sum to all $\mu _j$’s. The second step follows by integrating the estimate (2.2) on the horizontal segments of $\gamma$ and bounding the contribution of the vertical segments of the contour $\gamma$ by $\mathcal {O}(\eta _0)$. Finally, the third step is a consequence of the Stieltjes representation (2.3) and $|\mathfrak {R}_{\lambda ,t}(u)| \lesssim \Delta ^{-2}\lambda ^2$ for $|u| \gtrsim \Delta$. Using the estimate $\rho _0(u) = \rho _0(E_0) + \mathcal {O}(\Delta )$ for all $u\in I_{2\Delta }$ by admissibility of $E_0$ as in (2.4), we conclude that

3.80 $$\begin{aligned} \int _{I_{2\Delta }} \mathfrak {R}_{\lambda ,t}(u-\mu _k)\rho _0(u)\textrm{d}u =\rho _0(E_0) \int _{I_{2\Delta }} \mathfrak {R}_{\lambda ,t}(u-\mu _k)\textrm{d}u + \mathcal {O}(\Delta ), \end{aligned}$$

where we used $\int _\textbf{R}|\mathfrak {R}_{\lambda , t}(u)| \textrm{d}u \lesssim \lambda ^2 t\,\textrm{e}^{-2\alpha \lambda ^2 t} \lesssim 1$ as a consequence of (3.73). Moreover, a direct computation starting with (3.69) reveals that

3.81 $$\begin{aligned} \int _\textbf{R}\mathfrak {R}_{\lambda ,t}(u-\mu _k)\textrm{d}u = 0 \qquad \text {and} \qquad \int _{\textbf{R}\backslash I_{2\Delta }} \bigl |\mathfrak {R}_{\lambda ,t}(u-\mu _k)\bigr |\textrm{d}u \lesssim \frac{\lambda ^2}{\Delta } . \end{aligned}$$

Hence, combining estimates (3.76)–(3.81) yields

3.82 $$\begin{aligned} \bigl |\mathcal {R}\bigr |\lesssim \Delta + \eta _0+\lambda ^{-2}\epsilon _0+\Delta ^{-2}\lambda ^2 , \end{aligned}$$

which implies the $\mathcal {R}$-part of (2.20); the $\mathcal {E}$-part is an immediate consequence of Theorem 2.4 (a).

To complete the proof under the weaker assumption on $\langle \varvec{u}_j, A\varvec{u}_j\rangle$, stated in Footnote 6, we first *uniformly* approximate $\mathfrak {A}_N$ by a real analytic function $\mathfrak {A}_{N, \ell }: I_{2 \Delta } \rightarrow \textbf{R}$ with $\ell = \ell (\lambda , t)> 0$ (to be chosen below), which can be analytically extended to $\{z \in \textbf{C}: \textrm{dist}(z, I_{2 \Delta }) < \ell \}$ and satisfy $\sup _{N \in \textbf{N}}\Vert \mathfrak {A}_{N, \ell } - \mathfrak {A}_N \Vert _{\infty } \rightarrow 0$ as $\ell \rightarrow 0$. Such $\mathfrak {A}_{N, \ell }$ can be explicitly constructed, e.g., by convolution of $\mathfrak {A}_N$ with a Gaussian having variance of order $\ell$. For ease of notation, we shall now drop the subscript *N*. Then, the error term $\mathfrak {A} - \mathfrak {A}_\ell$ is easily seen to give a vanishing contribution (as $\ell \rightarrow 0$) by means of (3.75). Indeed, using (3.75) a bound analogous to (3.72), and $\Vert \mathfrak {p}\Vert _1 \le 1$, we find that

3.83 $$\begin{aligned} \biggl |\sum _{j,k}\frac{\mathfrak {p}_k}{r} \bigl (\mathfrak {A}(\mu _j) - \mathfrak {A}_\ell (\mu _j)\bigr ) \mathfrak {R}_{\lambda ,t}(\mu _j-\mu _k) \biggr |\lesssim \lambda ^2 t\,\textrm{e}^{-2\alpha \lambda ^2 t}\big (1 + \Delta + \lambda ^2/\Delta \big )\cdot \Vert \mathfrak {A} - \mathfrak {A}_\ell \Vert _\infty .\nonumber \\ \end{aligned}$$

Next, observe that using analyticity of $\mathfrak {A}_\ell$ and reasoning as in the proof for the case of $\mathfrak {A}$ being constant above, we obtain

3.84 $$\begin{aligned} \begin{aligned} \bigl |\mathcal {R}\bigr |\lesssim&~ \sup _{\mu _k\in I_\Delta }\biggl |\int _{I_{2\Delta }} \bigl (\mathfrak {A}_\ell (u)-\mathfrak {A}_\ell (\mu _k)\bigr )\mathfrak {R}_{\lambda ,t}(u-\mu _k)\textrm{d}u \biggr |\\  &+ \Delta + \eta _0+\lambda ^{-2}\epsilon _0+\Delta ^{ -2}\lambda ^2 + \big (1 + \Delta + \lambda ^2/\Delta \big )\cdot \Vert \mathfrak {A} - \mathfrak {A}_\ell \Vert _\infty . \end{aligned} \end{aligned}$$

Since $\mathfrak {A}_\ell (z)$ is analytic in the strip of width $\ell$, (3.73) implies that the integral on the right-hand side of (3.84) is bounded by

3.85 $$\begin{aligned} \frac{1}{\ell }\int _{I_{2\Delta }} \biggl (\frac{\lambda ^2 |u-\mu _k|}{|u-\mu _k|^2+(2\alpha \lambda ^2)^2} + \frac{t^{-1} |u-\mu _k|}{|u-\mu _k|^2+t^{-2}}\biggr )\textrm{d}u \lesssim \frac{\lambda ^2 |\log \lambda | + t^{-1} \log t}{\ell },\nonumber \\ \end{aligned}$$

uniformly in *k* such that $\mu _k \in I_\Delta$. Hence, choosing, say, $\ell := \lambda + t^{-1/2}$, this concludes the proof of Corollary 2.7. $\square$

### Microcanonical Average: Proof of Theorem 2.11

Using (2.12), we start by writing out3.86For the denominator, we have3.87from Lemma A.2. For the numerator, we use the assumption that  has uniformly bounded Lipschitz constant for $x \in I_\Delta$ (recall (2.27)). Hence we find

3.88 $$\begin{aligned} \int _\textbf{R}h(x) \, \langle \Im M_0(x + \textrm{i}\alpha \lambda ^2) \rangle _P \, \textrm{d}x = \pi h(E_0) + \mathcal {O}\big (\Delta + \lambda ^2 /\Delta \big ) \end{aligned}$$

completely analogously to (A.15) and (A.16), using Assumption 2.3.

Therefore, plugging (3.87) and (3.88) into (3.86), and using Assumption 2.2 (ii) together with Lemma A.2, we obtain

$$\begin{aligned} \langle A \rangle _{\widetilde{P}_\lambda } = \langle A \rangle _{{P}^\mathrm{(mc)}_{\lambda }} + \mathcal {O}(\mathcal {E}_{\textrm{mc}}) \quad \text {with} \quad \mathcal {E}_{\textrm{mc}}:= \epsilon _0 + \Delta + \lambda ^2/\Delta . \end{aligned}$$

This concludes the proof of Theorem 2.11. $\square$

## Appendix A. Auxiliary Results and Additional Proofs

### A.1 Auxiliary Results

In this subsection, we derive two technical lemmas, which are frequently used throughout the main text.

The first one (Lemma A.1) is concerned with properties of the self-consistent resolvent $M_\lambda (z)$ from the $\lambda$-dependent Quadratic Matrix Equation (3.1) using Assumption 2.2 on the unperturbed matrix $H_0$. Recall that $\frac{1}{6}\kappa _0$ denotes the upper bound for the energy width $\Delta$ (cf. Assumption 2.3).

#### Lemma A.1

Let $z:= E+\textrm{i}\eta$ be a spectral parameter in $\textbf{C}$ with $|z|\le C$, then the solution $M_{\lambda }(z)$ to (3.1) satisfies the bounds

A.1

Moreover, assuming that $\Im z \ge 0$, $|\Re z - E_0| \le \frac{1}{2}\kappa _0$, and $E_0$ lies in the admissible spectrum $\sigma _{\textrm{adm}}^{(\kappa _0, c_0)}$ of $H_0$ the following estimates

A.2 $$\begin{aligned} \Im m_\lambda (z)\gtrsim 1,\quad |m_\lambda (z)| \lesssim 1, \quad |m_\lambda '(z)| \lesssim 1, \end{aligned}$$

with  hold for any fixed $0 < \lambda \le \lambda _*$, and all $N \ge N_\lambda \in \textbf{N}$, uniformly in *z*.

#### Proof of Lemma A.1

First, we prove the (A.1) for $\Im z > 0$. Taking the imaginary part of the MDE (3.1), multiplying by $|M_\lambda |^2$ and taking the averaged trace yields

A.3

which immediately implies the first bound in (A.1). The second estimate in (A.1) follows from the first one by the Cauchy–Schwarz inequality. To extend (A.1) down to $\Im z = 0$, we first address the regularity^18^ of .

Differentiating the MDE (3.1), taking the trace, and invoking the first bound in (A.1), we obtain

A.4

where we used the imaginary part of (3.1) and the positive-definiteness of $\Im M_\lambda (z)$ to obtain the last inequality in (A.4). In the penultimate step, we used the first estimate in (A.1) to deduce the following chain of inequalities, dropping *z* for brevity,

A.5

Together with the first estimate in (A.1), (A.4) implies that $\lambda ^{2} \Im m_\lambda (z)$ is uniformly 1/3-Hölder continuous in $\{z\in \textbf{C}: |\Im z| \ge 0, |z| \le C\}$. Taking the limit $\Im z \rightarrow +0$ in (A.3), using (A.1) with $\Im z > 0$ and the regularity of $\lambda ^2 \Im m(z)$, we obtain the first bound in (A.1) with $\Im z = 0$. This concludes the proof of (A.1) for all $|z| \le C$ with $\Im z \ge 0$.

Next, we turn to proving the first estimate in (A.2). It follows from the MDE (3.1) that

A.6

and $$\Im z + \lambda ^2\Im m_\lambda (z) \ge \Im z$$.

First, assume that $\Im z \ge \eta _0$ (recall (2.2)). It follows from (A.1) that $\lambda ^2|m_\lambda (z)| \le \lambda$, hence by suitably shrinking the threshold $\lambda _*$, we can assume that $|\Re [z+\lambda ^2 m_\lambda (z)] - E_0| \le \frac{3}{4}\kappa _0$ for all *z* satisfying $\Im z \ge \eta _0$, $|z|\le C$ and $|\Re z - E_0| \le \frac{1}{2}\kappa _0$. Therefore

A.7 $$\begin{aligned} m_\lambda (z) = m_0\bigl (z+ \lambda ^2m_\lambda (z)\bigr ) + \mathcal {O}(\epsilon _0) = m_0(z) + \mathcal {O}(\lambda + \epsilon _0), \end{aligned}$$

where the first step follows by (2.2) and (A.1), and in the second estimate we used that $E_0 \in \sigma _{\textrm{adm}}^{(\kappa _0, c_0)}$, defined in (2.4). In particular, taking the imaginary part of (A.7), and using the positivity of $\rho _0$ in the admissible spectrum yields $\Im m_\lambda (z) \gtrsim 1 + \mathcal {O}(\lambda + \epsilon _0)$. Hence, from the 1/3-Hölder continuity of $\lambda ^{2}\Im m_\lambda (z)$, and (A.7) we deduce that

A.8 $$\begin{aligned} \lambda ^2\Im m_\lambda (z) \gtrsim \lambda ^2 + \mathcal {O}(\lambda ^3 + \lambda ^2\epsilon _0+ \eta _0^{1/3}). \end{aligned}$$

for all *z* with $|\Re z - E_0| \le \frac{1}{2}\kappa _0$, $\Im z \ge 0$, and $|z|\le C$. Therefore, for a suitably small threshold $\lambda _*$ and any fixed $0<\lambda \le \lambda _*$, the first estimate in (A.2) is established for all *N* satisfying $\eta _0(N)^{1/3} \lesssim \lambda ^2$ with the implicit constant depending only on the constant in (A.4). Since $\eta _0(N)$ converges to zero, there exists $N_\lambda \in \textbf{N}$ such that all $N\ge N_\lambda$ satisfy $\eta _0(N)^{1/3} \lesssim \lambda ^2$. Furthermore, we obtain $\Im z + \lambda ^2\Im m_\lambda (z) \gtrsim \lambda ^2 \ge \eta _0$, hence the second estimate in (A.2) follows from (2.2) and (A.6).

Finally, we prove the third estimate in (A.2). Differentiating (A.6) with respect to *z* yields

A.9

In particular, the first factor on the right-hand side of (A.9) is a normalized trace of $(H_0-\zeta )^{-2}$ with $\zeta := z + \lambda ^2m_\lambda (z)$, satisfying $|\Re \zeta - E_0| \le \frac{3}{4}\kappa _0$ and $\Im \zeta \ge 2c \lambda ^2 \ge \eta _0$ for some positive constant $c\sim 1$ by the first and second estimates in (A.2). Hence, integrating (2.2), we deduce that

A.10

Moreover, using the fact that $m_0(w)$ is the Stieltjes transform of the limiting density $\rho _0$, we conclude that

A.11

In particular, since $\Re \zeta$ lies in the admissible spectrum of $H_0$, the integral on the right-hand side of (A.11) admits the estimate

A.12 $$\begin{aligned} \biggl |\frac{1}{\pi }\int _{\textbf{R}} \frac{ \rho _0(x) }{(x-\zeta )^2}\textrm{d}x\biggr | = \biggl |\int _{J}\frac{\rho _0'(x) -\rho _0'(\Re \zeta )}{x-\zeta } \textrm{d}x\biggr | + \biggl |\int _{J} \frac{\rho _0'(\Re \zeta )\,\Im \zeta }{|x-\zeta |^2}\textrm{d}x \biggr | + \mathcal {O}(\kappa _0^{-2}),\nonumber \\ \end{aligned}$$

where $J:= [\Re \zeta -c\kappa _0, \Re \zeta +c\kappa _0] \subset I_{\kappa _0}$. Here we used that the kernel $(x-\Re \zeta )/|x-\zeta |^2$ is odd around $\Re \zeta$ and the integrability of $\rho _0$ to estimate the integral over $\textbf{R}\backslash J$, while the boundary term resulting from integration by parts over *J* is bounded by $\kappa _0^{-1}\mathcal {O}(\Vert \rho _0\Vert _{C^1(J)}) \lesssim \kappa _0^{-1}$. Observe that the second term on the right-hand side of (A.12) is bounded by $\mathcal {O}(|\rho _0'(\Re \zeta )|)$, while the first term is bounded by $\mathcal {O}(L)$, where *L* is the Lipschitz constant of $\rho _0'$ on the interval $[E_0-\kappa _0, E_0+\kappa _0]$. Therefore, . Finally, rearranging the identity (A.9) now yields

A.13

This concludes the proof of Lemma A.1.

We conclude this section by evaluating the denominator in (2.9).

#### Lemma A.2

Under Assumptions 2.2 and 2.3 (recalling the notation $\alpha = \pi \rho _0 (E_0)$) it holds that

A.14

#### Proof

We only prove the first relation in (A.14). The argument for the second estimate is analogous and hence omitted.

We have that

A.15

where we used $\int _\textbf{R}\langle \Im M_0(x +\textrm{i}\alpha \lambda ^2) \rangle _P \, \textrm{d}x = \pi$ and (2.2) to go to the second line. To go to the third line, we employed spectral decomposition (3.11) of $H_0$ and used Assumption 2.3. Next, using that $\mu _j \in I_\Delta$ and regularity of $\rho _0$ within $I_{2 \Delta }$, we can evaluate the integral in the last line of (A.15) as

A.16 $$\begin{aligned} \int _{\textbf{R}} \textrm{d}y \rho _0(y) \frac{1}{\pi }\frac{2\alpha \lambda ^2}{(y - \mu _j)^2 + (2\alpha \lambda ^2)^2} = \rho _0(E_0) + \mathcal {O}\big (\Delta + \lambda ^2/\Delta \big ), \end{aligned}$$

by adding and subtracting $\rho _0(E_0)$ and estimating $I_{2\Delta }$ and $I_{2 \Delta }^c$ separately.

Combining (A.15) with (A.16) and using that $\textrm{Tr}[P] = 1$ concludes the argument. $\square$

### A.2 Proof of Proposition 3.1

For simplicity of the presentation, we carry out the argument only for $\lambda = 1$; the claim in Proposition 3.1 follows from a natural rescaling. Our proof closely follows [12, Appendix B], [10, Section 5.2], and [9, Section 6.2],^19^ hence, we only give the main steps. Note that we are only interested in a *global law*, i.e. the spectral parameters $z_1, z_2$ have a distance to the spectrum of $H = D + W$ that is uniformly bounded from below by an *N*-independent positive constant (see also (3.5)). In particular, we can simply afford the norm bounds $\Vert G_i \Vert \lesssim 1$ and $\Vert M_i \Vert \lesssim 1$.

As a preparation for our argument, we recall the definition of the *second-order renormalization*, denoted by *underline*, from [11]. For functions $f(W), g(W)$ of the random matrix *W*, we define

A.17 $$\begin{aligned} \underline{f(W) W g(W)}:= &   f(W) W g(W) - \widetilde{\textbf{E}} \big [ (\partial _{\widetilde{W}}f)(W)\widetilde{W} g(W) \nonumber \\  &   + f(W) \widetilde{W} (\partial _{\widetilde{W}}g)(W) \big ], \end{aligned}$$

where $\partial _{\widetilde{W}}$ denotes the directional derivative in the direction of the GUE matrix $\widetilde{W}$ that is independent of *W*. The expectation is taken w.r.t. the matrix $\widetilde{W}$. Note that if *W* itself is a GUE matrix, then $\textbf{E}\underline{f(W)Wg(W)} = 0$, while for *W* with a general distribution, this expectation is independent of the first two moments of *W*. In other words, the underline renormalizes the product *f*(*W*)*Wg*(*W*) to the second order. We remark that underline (A.17) is a well-defined notation if the ‘middle’ *W* to which the renormalization refers is unambiguous. This is the case in our proof, since the functions *f*, *g* are resolvents, i.e. not involving explicitly monomials in *W*.

Moreover, we note that  and furthermore, that the directional derivative of the resolvent is given by $\partial _{\widetilde{W}} G = -G \widetilde{W} G$. For example, in the special case $f(W) = 1$ and $g(W) = (W + D -z)^{-1} = G$, we thus have

by definition of the underline in (A.17). Using this underline notation in combination with the identity $$G (W + D - z) = I $$ and the defining equation (3.1) for *M*, we have

A.18

Moreover, we have the following lemma, the proof of which is given at the end of this section.

#### Lemma A.3

(Representation as full underlined, cf. Lemma 5.6 in [10]). Under the notations and assumptions of Proposition 3.1, we have that

A.19

with $\mathcal {E}_{\textrm{iso}}:= 1/\sqrt{N}$ for some bounded deterministic matrix $B' \in \textbf{C}^{N \times N}$.

Having this approximate representation of the lhs. of (3.3) as a full underlined term at hand, we turn to the proof of (3.3) via a (minimalistic) cumulant expansion; see [12, Eq. (4.32)] and [10, Eq. (5.24)].

Let $p \in \textbf{N}$ be arbitrary. Then, abbreviating the lhs. of (A.19) by $\mathcal {Q}_{\varvec{x} \varvec{y}}$, we obtain

A.20 $$\begin{aligned}  &   \textbf{E}\big |\mathcal {Q}_{\varvec{x} \varvec{y}} \big |^{2p} \lesssim \, \textbf{E}\, \widetilde{\Xi } \, \big \vert \mathcal {Q}_{\varvec{x} \varvec{y}} \big \vert ^{2p-2} + \sum _{|\varvec{l}| + \sum (J \cup J_*) \ge 2}\textbf{E}\, \Xi (\varvec{l}, J, J_*) \big \vert \mathcal {Q}_{\varvec{x} \varvec{y}} \big \vert ^{2p-1 - | J \cup J_*|} + \mathcal {O}_\prec \big (\mathcal {E}_{\textrm{iso}}^{-2p}\big ) ,\nonumber \\ \end{aligned}$$

where the summation in (A.20) is taken over tuples $\varvec{l} \in \textbf{Z}^2_{\ge 0}$ and multisets of tuples $J, J_* \subset \textbf{Z}^2_{\ge 0} {\setminus } \{(0,0)\}$, for which we set $\partial ^{(l_1,l_2)}:= \partial _{ab}^{l_1} \partial _{ba}^{l_2}$, $|(l_1, l_2)| = l_1 + l_2$, $\sum J = \sum _{\varvec{j} \in J} |\varvec{j}|$. Moreover, we denoted

A.21 $$\begin{aligned} \widetilde{\Xi } :=\;&\frac{\left| \big ( G_1 {B}' G_1 B G_2\big )_{\varvec{x}\varvec{y}} \big ( G_1 G_2 \big )_{\varvec{x}\varvec{y}} \right| + \left| \big ( G_1 {B}' G_2 \big )_{\varvec{x}\varvec{y}} \big (G_1 B G_2 G_2 \big )_{\varvec{x}\varvec{y}}\right| }{N} \nonumber \\&+ \frac{ \left| \big ( G_1 B' G_2^*B^* G_1^*\big )_{\varvec{x}\varvec{x}} \big (G_2^* G_2\big )_{\varvec{y}\varvec{y}} \right| + \left| \big (G_1 B' G_1^*\big )_{\varvec{x}\varvec{x}} \big ( G_2^*A^* G_1^* G_2\big )_{\varvec{y}\varvec{y}}\right| }{N}\,, \nonumber \\ \end{aligned}$$

and defined $\Xi (\varvec{l}, J, J_*)$ via

A.22 $$\begin{aligned}&\Xi :=N^{-(|\varvec{l}| + \sum (J \cup J_*) + 1)/2} \sum _{ab} \big |\partial ^{\varvec{l}} \big [(G_1 {B}')_{\varvec{x}a}\big (G_2\big )_{b \varvec{y}} \big ]\big | \nonumber \\&\quad \times \prod _{\varvec{j} \in J} \big |\partial ^{\varvec{j}} \big (G_1 B G_2\big )_{\varvec{x}\varvec{y}}\big | \prod _{\varvec{j} \in J_*} \big |\partial ^{\varvec{j}} \big (G_2^* B^* G_2^*\big )_{\varvec{y}\varvec{x}} \big | \,. \end{aligned}$$

Now, by a simple norm bound, $\Vert G \Vert \lesssim 1$, we find that

A.23 $$\begin{aligned} \widetilde{\Xi } \prec \mathcal {E}_{\textrm{iso}}^2. \end{aligned}$$

For $\Xi$, our goal is to show that

A.24 $$\begin{aligned} \Xi (\varvec{l}, J, J_*) \prec \mathcal {E}_{\textrm{iso}}^{1 + | J \cup J_*|} . \end{aligned}$$

First, we have the naive bounds

A.25 $$\begin{aligned} \big |\partial ^{\varvec{l}} \big [(G_1 {B}')_{\varvec{x}a}\big (G_2\big )_{b \varvec{y}} \big ]\big | + \big |\partial ^{\varvec{j}} \big (G_1 B G_2\big )_{\varvec{x}\varvec{y}}\big | + \big |\partial ^{\varvec{j}} \big (G_2^* B^* G_2^*\big )_{\varvec{y}\varvec{x}} \big | \prec 1\nonumber \\ \end{aligned}$$

and hence

$$\begin{aligned} \Xi \prec N^{-(|\varvec{l}| + \sum (J \cup J_*) +1)/2} N^2&= N^{(4 - |\varvec{l}| )/2}N^{-(1+ \sum (J \cup J_*))/2} \\  &= N^{(4 - |\varvec{l}| )/2} \mathcal {E}_{\textrm{iso}}^{1+ \sum (J \cup J_*)} . \end{aligned}$$

Thus, for $|\varvec{l}| \ge 4$, we find the naive bounds (A.25) to be sufficient for (A.24), since trivially $| J \cup J_*| \le \sum (J \cup J_*)$. For $|\varvec{l}| \le 3$, we perform the summation $\sum _{ab}$ a bit more carefully, e.g., recalling the norm bound $\Vert G \Vert \lesssim 1$, as

$$\begin{aligned} \sum _{a} |G_{\varvec{x} a}| \le N^{1/2} \sqrt{\sum _{a} |G_{\varvec{x} a}|^2} = N^{1/2} (|G|^2)_{\varvec{x} \varvec{x}} \lesssim N^{1/2} \end{aligned}$$

instead of the naive $\sum _{a} |G_{\varvec{x} a}| \lesssim N$. Indeed, using the condition $|\varvec{l}| + \sum (J \cup J_*) \ge 2$, we can check all the cases $|\varvec{l}| \le 3$ explicitly and find

$$\begin{aligned}&\sum \limits _{ab} \big |\partial ^{\varvec{l}} \big [(G_1 {B}')_{\varvec{x}a}\big (G_2\big )_{b \varvec{y}} \big ]\big | \prod \limits _{\varvec{j} \in J} \big |\partial ^{\varvec{j}} \big (G_1 B G_2\big )_{\varvec{x}\varvec{y}}\big | \prod \limits _{\varvec{j} \in J_*} \big |\partial ^{\varvec{j}} \big (G_2^* B^* G_2^*\big )_{\varvec{y}\varvec{x}} \big |\\  &\quad \prec N^{2} \, N^{-(4 - |\varvec{l}| )/2}, \end{aligned}$$

from which we conclude (A.24).

Plugging (A.24) together with (A.23) into (A.20), using a Young inequality and recalling that *p* was arbitrary, we deduce that

$$\begin{aligned} |\mathcal {Q}_{\varvec{x} \varvec{y}}| \prec \mathcal {E}_{\textrm{iso}} = \frac{1}{\sqrt{N}}, \end{aligned}$$

i.e. we have proven Proposition 3.1. $\square$

It remains to give the proof of Lemma A.3.

#### Proof of Lemma A.3

Applying (A.18) to $G_2$, we thus find that

for $\widetilde{B} = \mathcal {X}_{12}[B]$, where we introduced the linear operator

A.26

Extending the underline to the whole product, we obtain

from which we conclude

A.27

To proceed, we note that

A.28

has bounded norm, $\Vert B' \Vert \lesssim 1$, since for $z_1, z_2$ away from the spectrum of *H*, $\mathcal {X}_{12}$ is a bounded operator (see [10, Lemma B.5] and [9, Appendix A.2]). This can be seen as follows: First, the denominator in (A.28) is estimated as . Next, we employ the identity , as follows from the MDE (3.1) and, together with a Schwarz inequality, implies boundedness of the numerator in (A.28). Finally,  follows from the fact that  in the regime of $z_i$’s with distance to the spectrum of *H* bounded below by an *N*-independent positive constant.

Then, using the norm bounds $\Vert G_i \Vert \lesssim 1$ and $\Vert M_i \Vert \lesssim 1$ together with the *single resolvent global law* (3.2) (see also [22, Theorem 2.1]) for the second, fourth and fifth term in (A.27), we conclude the desired.

## Footnotes

1 Unperturbed systems $H_0$, for which the time evolution $\langle A \rangle _{P_0(t)}$ does not approach the microcanonical prediction of equilibrium statistical mechanics, but nevertheless have a large-*t* limit (a non-thermal steady state), are studied in [5]. In a sense, these systems exhibit prethermalization, although they do not approach thermal equilibrium after the first steady state is reached.

2 A careful examination of our proof reveals that the entries of *W* need not be distributed identically. Indeed, only the matching of the second moments is necessary, but higher moments can differ.

3 Here, $C^{1,1}(J)$ denotes the set of continuously differentiable functions with a Lipschitz-continuous derivative on an interval *J*, equipped with the norm $\Vert f\Vert _{C^{1,1}(J)}:= \Vert f\Vert _{C^1(J)} + \sup \limits _{x,y\in J: x\ne y} \frac{|f'(x)-f'(y)|}{|x-y|}$.

4 Recall that the imaginary part of a matrix $B\in \textbf{C}^{N\times N}$ is given by $\Im B=\frac{1}{2i}(B-B^*)$.

5 We use the convention that the Fourier transform of $f \in L^1(\textbf{R})$ is defined as $\widehat{f}(p):= (2 \pi )^{-1/2} \int _{\textbf{R}} f(x) \textrm{e}^{-\textrm{i}px} \textrm{d}x$.

6 In fact, it is sufficient to assume that the overlaps are regular in *j* in the sense that there exists a uniformly equicontinuous sequence $(\mathfrak {A}_N)_{N \in \textbf{N}}$ of functions $\mathfrak {A}_N: I_{2 \Delta } \rightarrow \textbf{R}$ such that

$$\begin{aligned} \langle \varvec{u}_j, A \varvec{u}_j \rangle = \mathfrak {A}_N(\mu _j) + \mathcal {O}\big (\mathcal {E}_{\textrm{LOR}}\big ) \quad \text {for all} \quad j \in \textbf{N}\quad \text {with} \quad \mu _j \in I_{2\Delta }. \end{aligned}$$

Alternatively, we could also assume that, for every fixed $\Delta > 0$ small enough, the overlaps $\langle \varvec{u}_j, A \varvec{u}_j \rangle$ are well approximated by $\mathfrak {A}_N(\mu _j)$ in $\ell ^p$-sense for some $p\ge 1$, i.e.,

$$\begin{aligned} \frac{1}{N}\sum _{j:\mu _j\in I_{2\Delta }} \left| \langle \varvec{u}_j, A \varvec{u}_j \rangle -\mathfrak {A}_N(\mu _j)\right| ^p \overset{N\rightarrow \infty }{\longrightarrow }0 . \end{aligned}$$

The case $p=2$ is reminiscent of the so-called *weak ETH* studied in [7, 16].

7 A common variant of this requirement in the physics literature is to assume the validity of (2.24a) only for *local* observables, i.e. supported in a finite region of an underlying space (see, e.g., [29, Section 5.2]).

8 We use the usual notation for the commutator, i.e. $[A,B]:= AB - BA$ for all matrices *A*, *B*.

9 Indeed, taking, say, $E_\lambda \in I_{\Delta /2}$ it can be rigorously shown that the difference $\langle \varvec{v}_{\lambda } A \varvec{v}_{\lambda } \rangle - \langle A \rangle _{P^\mathrm{(mc)}_{\lambda }}$ vanishes in the triple limit (2.11). More precisely, this follows from the *Eigenstate Thermalization Hypothesis (ETH)* for the random matrix $H_\lambda = H_0 + \lambda W$ (see [9, Theorem 2.6]) and using assumption (2.27) together with (3.1) and (A.7).

10 In the sequel, we consistently use the letter *G* to denote resolvents of random matrices, for example $G_\lambda (z):= (H_\lambda - z)^{-1}$, and *M* to denote deterministic resolvents – more specifically, resolvents of $H_0$. .

11 The MDE in the context of mean-field random matrices was introduced in [2] and extensively analyzed in [3].

12 In fact, by examining the proof of Proposition 3.1, it can easily be seen that the dependence on both small parameters $\lambda$ and *c* is at most (inverse) polynomial.

13 Note that (3.6) is an *averaged* two-resolvent local law, but we prove it via an *isotropic* law. Therefore, the error term in (3.6) is not optimal: The true bound is of order $N^{-1}$ instead of $N^{-1/2}$. While we could have obtained an optimal error term with the same proof idea, the weaker bound is sufficient for our analysis and we use it for simplicity.

14 Recall that the flat pieces of the contours $\gamma _1$ and $\gamma _2$ from Fig. 3 lie on the lower and upper half plane, respectively.

15 Here and in the following, $|\textrm{d}z|$ denotes the total variation of the complex measure $\textrm{d}z$.

16 The integral in (3.45) diverges logarithmically as *x* approaches the intersection of the contour $\gamma _2$ with the real line. However, the contribution of such singularities to the $\textrm{d}x$ integral on the right-hand side of (3.44) is negligible.

17 Inequality (3.72) can be interpreted as a discrete analog of Young’s convolution inequality, which cannot be evoked directly since the eigenvalues $\mu _j$ do not form a group under addition.
